# Synthesis, characterization, PXRD studies, and theoretical calculation of the effect of gamma irradiation and antimicrobial studies on novel Pd(II), Cu(II), and Cu(I) complexes

**DOI:** 10.3389/fchem.2024.1357330

**Published:** 2024-02-12

**Authors:** Safaa S. Hassan, Samar A. Aly, Ahlam I. Al-Sulami, Salwa A. H. Albohy, Mohamed F. Salem, Ghada M. Nasr, Ehab M. Abdalla

**Affiliations:** ^1^ Chemistry Department, Faculty of Science, Cairo University, Giza, Egypt; ^2^ Department of Environmental Biotechnology, Genetic Engineering and Biotechnology Research Institute, University of Sadat City, Sadat, Egypt; ^3^ Department of Chemistry, College of Science, University of Jeddah, Jeddah, Saudi Arabia; ^4^ Chemistry Department, Faculty of Science (Girls), Al-Azhar University, Nasr, Cairo, Egypt; ^5^ Department of Molecular Diagnostics and Therapeutics, Genetic Engineering and Biotechnology Research Institute, University of Sadat City, Sadat, Egypt; ^6^ Chemistry Department, Faculty of Science, New Valley University, El-Kharga, Egypt

**Keywords:** complexes, γ-irradiation, antimicrobial, thermal, DFT, molecular docking

## Abstract

The main objective of this study is to synthesize and characterize of a new three complexes of Pd (II), Cu (II), and Cu (I) metal ions with novel ligand ((Z)-2-(phenylamino)-N'-(thiophen-2-ylmethylene)acetohydrazide) H_2_L_B_. The structural composition of new compounds was assessed using several analytical techniques including FT-IR, ^1^H-NMR, electronic spectra, powder X-ray diffraction, and thermal behavior analysis. The Gaussian09 program employed the Density Functional Theory (DFT) approach to optimize the geometry of all synthesized compounds, therefore obtaining the most favorable structures and crucial parameters. An investigation was conducted to examine the impact of γ-irradiation on ligands and complexes. Before and after γ-irradiation, the antimicrobial efficiency was investigated for the activity of ligands and their chelates. The Cu(I) complex demonstrated enhanced antibacterial activity after irradiation, as well as other standard medications such as ampicillin and gentamicin. Similarly, the Cu(I) complex exhibited superior activity against antifungal species relative to the standard drug Nystatin. The docking investigation utilized the target location of the topoisomerase enzyme (2xct) chain A.

## 1 Introduction

Hydrazone derivatives are a large class of chemicals used in various medicinal applications (Abdalla et al., 2021) because of their broad range of pharmacokinetic features (Popiołek et al., 2018; Aneja et al., 2019; El-saied et al., 2020; Katariya et al., 2020), particularly in drug detection programmers. The hydrazone and carbaldehyde derivatives, as well as their complexes, have been reported to reveal a wide range of biological features (Sepay and Dey, 2014; Parveen et al., 2018; Naveen et al., 2020; Zülfikaroğlu et al., 2020), such as anticancer (Mandewale et al., 2017; Manohar et al., 2018; Yousefi et al., 2019; Babahan et al., 2020), antibacterial (Ekennia et al., 2018; Özbek et al., 2019; Khan et al., 2020), antimicrobial (Cao et al., 2018; Philip et al., 2018; Santiago et al., 2020), antifungal (Rocha et al., 2019; Elsayed et al., 2020), antimalarial (Maurya et al., 2017), antiviral (Sreepriya et al., 2020), antimycobacterial (Mandewale et al., 2017; Mandewale et al., 2018), antileishmanial (Coimbra et al., 2019), antiplatelet (Margariti et al., 2020), anti-analgesic antitubercular, anticonvulsant (Dehestani et al., 2018), anti-uropathogenic (Alodeani et al., 2015), antiproliferative (Bergamini et al., 2019), antiarthritic (Shabbir et al., 2014), and antioxidant properties (Vanucci-Bacqué et al., 2016; Anastassova et al., 2018; Al-Hazmi et al., 2020); potent immunomodulatory agents (Meira et al., 2018); and potent antiangiogenic agents in atherosclerosis (Vanucci-Bacqué et al., 2016). They are also essential in Alzheimer’s disease treatment (Haghighijoo et al., 2017; Parlar et al., 2019).

Garoufis et al. evaluated several scholarly studies on the antibacterial, antiviral, and antifungal properties of Pd II) chelates with diverse ligands (Other ligands include nitrogen and sulfur donor sites, Schiff base ligands, and other medications) (Guerra et al., 2005; Garoufis et al., 2009). Other studies have recently been published in the literature that revealed diverse palladium complex activity intensities on distinct bacteria and fungus species (Aghatabay et al., 2007; Biyala et al., 2008; Vieira et al., 2009).

Copper is the primary component of copper doorknobs and touch surfaces in hospitals and healthcare facilities that prevent bacteria and diseases from growing and spreading (“contact killing") (Tian et al., 2012). Many copper complexes with potential and diverse biological action have been discovered in the literature, including antibacterial (Beeton et al., 2014; Lobana et al., 2014), anticancer (Qiu et al., 2015; Stefani et al., 2015), anticonvulsant (Lemoine et al., 2002), antifungal (Soroceanu et al., 2016), anti-inflammatory (Hoonur et al., 2010), antimalarial (Hubin et al., 2014), anti-neurodegenerative (Quintanova et al., 2015), antiobesity (Perontsis et al., 2016), antioxidant (Tolia et al., 2013), anti-rheumatic (Sherif and Hosny, 2014), antithyroid (Urquiza et al., 2015), antitubercular (Netalkar et al., 2014), and antiviral activity (Dorotíková et al., 2015).

Radiation is widely used in biomaterials science to alter surface properties, clean surfaces, and improve bulk properties. In addition to biochips and situ photopolymerizable bioglues, radiation is used to develop biochips (Aly and Elembaby, 2020b). Gamma radiation, great-energy electrons, and ultraviolet radiation are the most common energy sources used to irradiate biomaterials (Balashova et al., 2019; Aly et al., 2022a; Elganzory et al., 2022). The manuscript aimed to prepare, characterize, and investigate the impact of gamma irradiation on Pd(II), Cu(II), and Cu(I) metal complexes and also an antimicrobial study of these compounds.

## 2 Experimental method

### 2.1 Synthesis of hydrazone ligand

First, 20 mL of ultra-grade ethanol was added to a round flask along with 0.01 mol of 2-(p-tolylamino)acetohydrazide and 0.01 mol of 5-hydroxy-4-oxo-4H-pyran-3-carbaldehyde. The resultant liquid was mixed for approximately 6 h at room temperature (Abdalla et al., 2021). The resulting precipitate was separated after washing and drying into a filter paper. The washing solutions were ethyl alc and diethyl ether (Aly et al., 2021).

C_15_H_15_N_3_O_4_ (H_2_L_B_): Yellow, Molecular Weight: 301.3, Yield = 94%; Analytical Calculated: H, 5.02; C, 59.80; N, 13.95. Exp. (%):H, 4.98; C, 59.76; N, 13.92. The FTIR bands (cm^-1^) at 3,392, 3,202, 1,677, 1,639, and 1,604 related to (OH/H_2_O), (N-H), (C=O)_side,_ and (C=N) respectively. Electronic transitions: λ_max_: 341, 399. ^1^HNMR: δ (ppm): 2.10, 3.91, 5.84–6.63, 7.11, 11.10, and 15.90 were related to (s, 3H, CH_3_), (s, 2H, NCH_2_), (s, 1H, NH), (s, 1H, NCH), (m, 4H, p-sub. Ph-H), (s, 2H, pyran-H), (bs, 1H, NHC = O) and (bs, 1H, OH) respectively. ^13^CNMR: δ (ppm): 19.2, 45.3, 148.2,(116.7, 128.7, 163.1, 169.1, 170.5), (112.4, 116.4, 128.8, 148.1) and 181.5 corresponded to (CH_3_), (NCH_2_), (C=N), (pyran-C), (Ph-C) and (C=O) respectively. Supplementary Figure S2.

### 2.2 Synthesis of metal complexes

**SCHEME 1:**
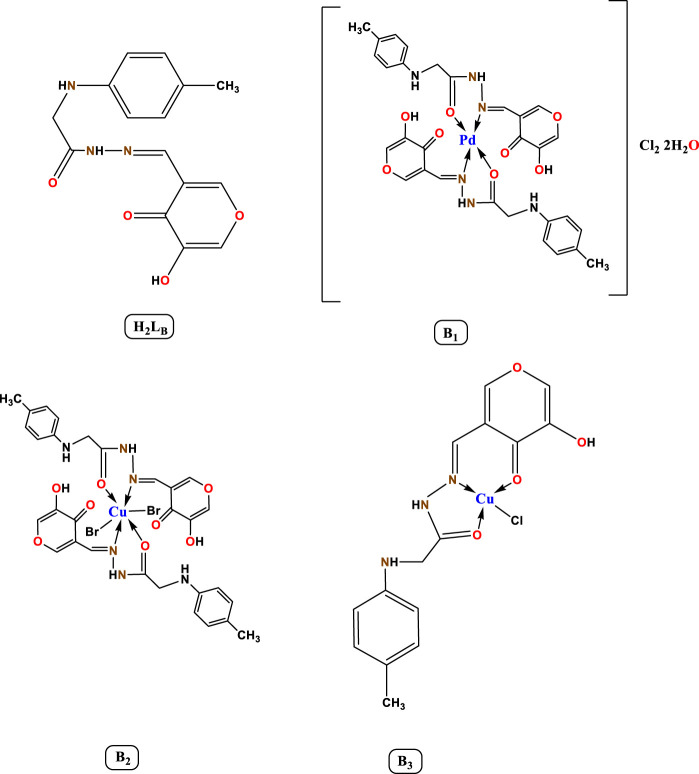
The proposed structures of hydrazone ligands and their metal chelates.

According to a standard approach (Scheme 1), the metal salt was added to the ligand with a stoichiometric quantity (1 mmol; = 0.177 g Pd II); 0.099 g Cu(I); 0.223 g Cu(II) to 1 mmol = 0.301 of ligand). The solvent used was EtOH (20 mL). The reaction mixture was refluxed for 6 hours while stirring at 60°C. The colorful reaction product that resulted from the reaction was filtered out of the reaction mixture, extensively cleaned with ethyl alc to eliminate the unreacted starting residues, and vacuum-dried (Frisch et al., 2009a). TLC was used to verify the complexes' purity.

### 2.3 Analytical and physical measurements

All of the starting materials used in this investigation (which were of analytical quality and were not purified before use) were provided by Sigma-Aldrich and Fluka. Detailed information on the tools and procedures used for structure confirmation and application are provided in the Supplementary Material.

### 2.4 Quantum chemical calculation QCC

GaussView 5.0.8 (Wallingford, CT 2009) (Frisch et al., 2009a) was used to prepare the input files of compounds. Gaussian 09 rev. A.02 was used for all computations (Wallingford, CT, 2009) (Frisch et al., 2009b). The DFT/B3LYP technique was used. The standard basis sets were 6311G and LANL2DZ concerning the synthesized H_2_L and its metal complexes, respectively (Ammar et al., 2014; Maurya et al., 2015; Ding et al., 2018; Abdalrazaq et al., 2022; Aly et al., 2022b; Mahani and Mohammadi, 2022; Ugurlu and Harmankaya, 2022). The topoisomerase IIa was simulated using MOE 2009 (Molecular Operating Environment) software. The topoisomerase II DNA gyrase protein crystal structure (PDB ID: 2XCT) was downloaded from the protein data bank. The docking steps involved constructing the investigated compounds using Chembio3D-ultra software followed by their optimization. The selected protein was downloaded from Protein Data Bank PDB. Finally, the binding affinity of each compound to the protein was evaluated.

### 2.5 Assay for antimicrobials

The antibacterial activity of the generated compounds was evaluated using the agar well diffusion method as reported in detail (Abdalla et al., 2020; Al-Farhan et al., 2021) and specifics of the antimicrobial screening process are provided in the Supplementary Material.

### 2.6 Irradiation studies

Solid ligand and chelate complex samples were submitted to irradiation tests at a dosage of 60 kGy (Aly and Elembaby, 2020a) Supplementary Section S3.

## 3 Result and discussion

### 3.1 Characterization

The chelates' analytical results confirmed the predicted chemical formulae and demonstrated the formation of 1:2 of Pd(II) and 1:1 of Cu(II) and Cu(I) (M:L) (Table 1). The molar conductance values were measured in 10^−3^ M DMF for the Cu (II), Cu(I), and Pd(II) complexes to give 25, 30, and 65 Ω^-1^cm^2^mol^-1^ respectively. This result revealed that the Cu(II) and Cu(I) chelates were non-electrolytic (Aly and Fathalla, 2020); on the other hand, the Pd(II) complex was electrolytic.

**TABLE 1:** Physicochemical parameters of all compounds.

No.	Compounds Molecular Formula	M.Wt	Color Yield %	^∧^m	µ_eff_/BM	(Cal.) Found %			
						C	H	N	M
H_2_L_B_	C_15_H_15_N_3_O_4_	301.30	Yellow 74	—	—	59.80 (59.76)	5.02 (4.98)	13.95 (13.92)	—
B_1_	Pd(H_2_L)_2_]Cl_2_.2H_2_O C_30_H_34_Cl_2_N_6_O_10_Pd	815.95	Orange 70	65	Dia	44.16 (44.03)	4.20 (4.11)	10.30 (10.21)	13.04 (12.96)
B_2_	Cu(H_2_L)_2_Br_2_ C_30_H_30_Br_2_CuN_6_O_8_	825.96	Green 76	25	1.74	43.63(43.57)	3.66 (3.58)	10.18 (10.09)	7.69 (7.61)
B_3_	Cu(H_2_L)Cl C_15_H_15_ClCuN_3_O_4_	400.30	Green 72	30	Dia	45.01 (44.96)	3.78 (3.74)	10.50 (10.43)	15.87 (15.82)

Where: ^∧^m = molar conductivity (ohm^-1^, cm^2^ mol^-1^) in 10^−3^ M DMF, solution.

### 3.2 ^1^H- NMR interpretation of the non-irradiated and radiated ligands

(H_2_L) ^1^H-NMR spectra in DMSO-_d6_ (defining each ligand before and after irradiation) were confirmed. After radiation exposure, the signals stayed in the same spot or slightly moved in the ligand’s ^1^H-NMR spectrum (Supplementary Figure S1). Even yet, the bands' strength after irradiation was greater than before. The ^1^H-NMR spectrum of the NH proton had a singlet signal at 5.94 ppm and a wide single peak at 11.1 ppm that identified the NHC = O proton in the hydrazone linkage. The spectral signature of aromatic ring protons also included a multiple signal between 6.70 and 6.75 ppm. The spectrum also exhibited singlet signals at 7.11 ppm, which were pyrene proton signals, and a wide single peak at 15.90 ppm, a hydroxyl group C-OH proton signal.

### 3.3 FT-IR spectra

Table 2 and Supplementary Figures S3-S6 clarify the IR spectra of the irradiated (A) and non-irradiated (B) states of H_2_L and its metal chelates. The IR spectra of H_2_L_B_ and H_2_L_A_ have revealed bands at 3,392, 3,391; 1,677, 1,675; 1,639, 1,635; and 1,604, 1,600 cm^-1^ which corresponded to the Hydroxyl group, ν(carbonyl) _side, ring_, and ν(C=N) for the non-irradiated and irradiated states respectively. Upon complexation, these bands were shifted to a higher or lower value where the band related to ν(OH) was shifted to higher values as in the Pd(II), Cu(II), and Cu(I) chelates exhibited at 3,435, 3,434; 3,440, 3,490 and 3,490, 3,487 cm^-1^, respectively. The ν (C=N) bands were changed at higher frequency after irradiation and were displayed at 1,545, 1,547; 1,550, 1,548; and 1,550 cm^-1^, respectively. The bands exhibited at 1,687, 1,685; 1,696, 1,698; and 1,695, 1,693 cm^-1^ related to ν(OH) and ν(C=O)_side_ respectively were changed to a higher frequency, while the bands of ν(C=O)_ring_ and ν(C=N) were changed to a lower frequency. New signals were seen in the region at 603, 600; 537, 536; and 537, 579 cm^-1^ related to ν (M-Oxygen) and ν(M-Nitrogen) were seen in the region at 539, 545; 485, 479; and 508, 513 cm^-1^ respectively (Elganzory et al., 2022). The infrared spectra for irradiated H_2_L and chelates revealed changes in the size and intensity of the bands with the action of irradiation in all compounds (Abdalla et al., 2021). Supplementary Figure S7 shows the theoretical FT-IR spectra powder pattern of ligand and its complexes.

**TABLE 2:** FT- IR spectral bands all compounds before (B) and after (A) irradiation in 4,000–400 cm^-1^, A = After irradiation and B = Before irradiation.

No.	Compound	ν(OH/H_2_O)	ν(N–H)	ν(C=O)_side_	ν(C=O)_ring_	ν(C=N)	ν(M-O)	ν(M-N)
H_2_L_B_ H_2_L_A_	C_15_H_15_N_3_O_4_	3,392 3,391	3,202 3,202	1,677 1,675	1,639 1,635	1,604 1,600	—	—
DFT		3,595	3,244	1700	1,633	1,597	—	—
B_1_ A_1_	[Pd(H_2_L)_2_]Cl_2_.2H_2_O	3,435 3,434	2,921 2,917	1,687 1,685	1,625 1,621	1,545 1,547	603 600	539 545
DFT		3,424	3,046	1,669	1,642	1,552	598	535
B_2_ A_2_	Cu(H_2_L)_2_Br_2_	3,440 3,490	3,251 3,205	1,696 1,698	1,601 1,600	1,550 1,548	537 536	485 479
DFT		3,487	3,280	1,696	1,624	1,561	535	490
B_3_ A_3_	Cu(H_2_L)Cl	3,490 3,487	3,188 3,191	1,695 1,693	1,602 1,601	1,550 1,550	537 579	508 513
DFT		3,500	3,199	1,687	1,597	1,552	589	517

### 3.4 Mass spectra

Pd(H_2_L)_2_]Cl_2_.2H_2_O, Cu(H_2_L)_2_Br_2_, and Cu(H_2_L)Cl showed molecular ion peaks at m/z 815.11 (21%), 825.42 (12%), and 399.62 (32%), These findings were supported by the proposed molecular formulae that have been provided (calc. 815.95, 825.96, and 400.30 amu), respectively (Supplementary Figures S8-S10).

The mass fragmentation patterns of complexes are shown in Scheme S1-S3, where the mass spectra’s multi-peaks pattern resulted in a sequence of peaks corresponding to the distinct fragments.

### 3.5 Electronic spectra

Table 3 and Supplementary Figures S11, S12 show all compounds' UV–Vis spectra before and after irradiation at room temperature in a 10^−3^ DMF solution in the 200–800 nm range. In the UV range, the absorption spectra of H_2_L_B_ and H_2_L_A_ showed two absorption bands (Liu et al., 2013); the first bands were seen at λ_max_ = 341, 335 nm related to π–π* transition, and the second transitions were seen at λ_max_ = 399, 394 nm assigned to n–π* transition. The Pd(II) chelates (B_1_ and A_1_) explored bands at 332; 326, 393; 389 and 511; 502 nm, assigned to ^1^A_1g_ → ^1^B_1g_ transition representing a square planar geometry (El-Boraey and El-Gammal, 2015; Shankarwar et al., 2015). The electronic spectra of Cu II) complexes (B_2_ and A_2_) revealed bands at 352, 346; 399, 394; and 571, 563 nm, pointing to n–π* and d-d transition representing octahedral geometry. Moreover, Cu(I) chelates(B_3_ and A_3_) before and after irradiation revealed four bands at 343, 331; 399, 397; 487, 469; and 615, 595 nm, respectively, pointing to n–π* and d-d transition representing tetrahedral geometry. The difference between the electronic transitions of the H_2_L and all chelates was noted after irradiation as the λ_max_ position and the absorbance value (Abdalla et al., 2021).

**TABLE 3:** Electronic spectra data of all compounds before (H_2_L_B_, B_1_- B_3_) and after (H_2_L_A_, A_1_- A_3_) irradiation.

No	Compound	λ_max_(nm)	DFT
H_2_L_B_ H_2_L_A_	H_2_L	341, 399 335, 394	310,430
B_1_	Pd(H_2_L)_2_Cl_2_.2H_2_O	332,393, 511	650
A_1_		326,389, 502	
B_2_	Cu(H_2_L)_2_Br_2_	352, 399, 571	620
A_2_		346, 394, 563	
B_3_	Cu(H_2_L)Cl	343, 399, 487, 615	450, 460, 600
A_3_		331, 397, 469, 595	

### 3.6 X-ray diffraction

**FIGURE 1:**
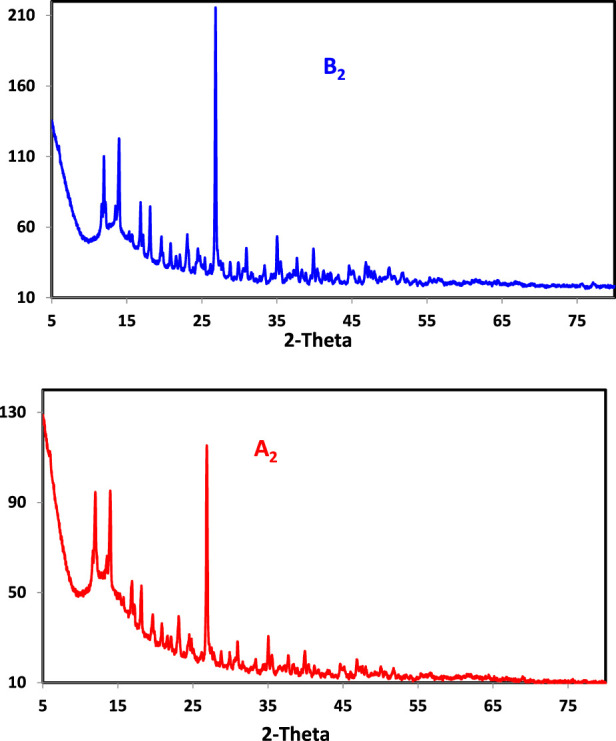
PXRD powder pattern of Cu (II) complexes in non-irradiated (B) and irradiated (A) states.

PXRD was used when the single crystals of the produced chelates failed to grow. Before and after irradiation, powder diffraction patterns of ligands (H_2_L_B_, H_2_L_A_) and Cu(II) complexes (B_2_) and (A_2_) were recorded across 2θ = 5°–80° (Table 4; Figure 1, Supplementary Figure S13). The greatest intensity peak was also identified, along with its location, half-maximum width, and *d* spacing. The reflection in the H_2_L diffractogram peaks was shown at 2θ = 15.822° and 15.801, which corresponded to *d* values of 0.559119 and 0.558271 nm, respectively. Coordination compounds have been generated because the powder XRD patterns of the ligands (H_2_L_B_, H_2_L_A_) and the complexes (B_2_ and A_2_) were completely different. The patterns exhibited well-characterized crystalline peaks, indicating that the ligand was in crystallized form in the (B_2_ and A_2_) and (H_2_L_B_, H_2_L_A_) complexes. The average particle sizes of (H_2_L_B_, H_2_L_A_) and (B_2_ and A_2_) chelates were calculated using the Scherer equation (Muniz et al., 2016; Abdalla et al., 2020; Abdel Rahman et al., 2022; Abdel-Rahman et al., 2022) and were 37.48; 31.99 and 51.64; 40.25 nm, respectively. The crystal size variation might be due to the Nano range caused by the irradiation.

**TABLE 4:** PXRD data of ligand and chelates (B) before and (A) after irradiation.

Compound	Angle 2θ	d- value nm	FWHM	Grain size Nm
H_2_L_B_	15.822	0.559119	0.238	37.48
H_2_L_A_	15.801	0.558271	0.262	31.99
B_2_	26.321	0.332158	0.167	51.64
A_2_	26.400	0.331720	0.213	40.25

### 3.7 Thermal studies

The thermal TGA of all compounds before (H_2_L_B_, B_1_-B_3_) and after (H_2_L_A_, A_1_-A_3_) irradiation were investigated with a heating range from (20°C–800°C). Table 5 and Supplementary Figure S14.

**TABLE 5:** Thermal data of all compounds before (B) and after (A) irradiation.

Compound		Temp. range/^o^C TGA	Mass loss% Calc.F)	Leaving species
C_15_H_15_N_3_O_4_	H_2_L_B_	197–250	51.4 (51.18)	C_6_H_6_N_2_O_3_ C_4_H_4_ C_5_H_5_NO
Residue		250–430	17.27 (17.30)	
		>430	31.55 (31.51)	
C_15_H_15_N_3_O_4_	H_2_L_A_	197–250	40.59 (40.53)	C_6_H_6_N_2_O C_4_H_4_NO_2_ C_5_H_5_O
Residue		250–501	32.51 (32.55)	
		>501	26.87 (26.91)	
C_30_H_34_Cl_2_N_6_O_10_Pd	B_1_	45–225	13.27 (13.35)	2H_2_O+2HCl
Residue	A_1_	225–386	71.59 (71.64)	C_30_H_28_N_6_O_7_
			14.94 (15.00)	PdO
		>386	13.32 (13.35)	
			71.61 (71.64)	
			14.97 (15.00)	
C_30_H_30_Br_2_CuN_6_O_8_	B_2_	30–210	19.53 (19.59)	2HBr
	A_2_	210–305	23.13 (23.03)	C_10_H_12_N_3_O
		305–591	47.71 (47.74)	C_20_H_16_N_3_O_6_
		>591	9.60 (9.63)	CuO
Residue		30–191	9.76 (9.79)	HBr
		191–461	34.91 (34.88)	C_10_H_14_N_3_O_2_Br
		461–610	47.53 (47.50)	C_20_H_14_N_3_O_6_
		>610	7.65 (7.69)	Cu
C_15_H_15_ClCuN_3_O_4_	B_3_	100–198	9.09 (9.12)	HCl
		198–495	38.21 (38.25)	C_6_H_5_N_2_O_3_
Residue	A_3_	495–571	36.83 (36.76)	C_9_H_9_NO
		>571	15.84 (15.87)	Cu

#### 3.7.1 Pd(II) chelates under the two irradiated and unirradiated cases

The TGA curves of chelates (B_1_ and A_1_) (Supplementary Figure S14) showed three steps for losing weight (Calc./Exp.%: 13.35/13.27 and 13.35/13.32), assigned to the release of two hydrated water molecules and two hydrogen chloride molecules within the heat at 45–225°C. The second step showed the appearance of the decomposition within the heat from 225 to 386°C with losing weight (Calc/Exp.%: 71.64/71.59 and 71.64/71.61) and corresponded to C_30_H_28_N_6_O_7_ leaving PdO as a residue (Aly and El-Boraey, 2019).

#### 3.7.2 Cu(II) chelates under the two irradiated and unirradiated cases

The TGA curves of the chelates (B_2_ and A_2_) were four steps apart (Supplementary Figure S14), where weight loss was shown in the temperature ranges of 30–210 and 30–191°C (Calc./Found percent: 19.59/19.53 and 9.79/9.76) which agreed to the loss of two and one molecules of hydrogen bromide. The decomposition of (B_2_ and A_2_) was within the heating range 210–305 and 191°C–461°C with weight loss (Calc./Exp.%: 23.03/23.13 and Calc./Found%: 34.88/34.91), which corresponded to the dissociation of C_10_H_12_N_3_O and C_10_H_14_N_3_O_2_Br. The third step in the range 305–591 and 461°C–610 °C indicated the removal of C_20_H_16_N_3_O_6_ and C_20_H_14_N_3_O_6_ with weight loss (Calc./Found%: 47.74/47.71 and 47.50/47.53). The final step over 591°C and 610 °C indicated the removal of CuO and Cu as the final remainder from unirradiated (B_2_) and irradiated Cu(II) complexes(A_2_), respectively (El-Boraey and Mansour, 2018).

#### 3.7.3 Cu(I) chelates under the two irradiated and unirradiated cases

The TGA curves of chelates (B_3_ and A_3_) (Supplementary Figure S14) showed similar four steps within the heat range 100–19°C, which estimated the loss of hydrogen chloride molecule with mass loss (Calc./Found%: 9.12/9.09). The second step showed the mass loss (Calc./Found%: 38.25/38.21) in a temperature range of 198–495°C, corresponding to the losses of C_6_H_5_N_2_O_3_. The decomposition stage of (B_3_ and A_3_) through temperature range 495–571°C providing mass loss (Calc./Exp.%: 36.76/36.83) indicated the removal of C_9_H_9_NO, leaving copper metal as final residue. Finally, the thermal stability of the irradiated complexes using gamma rays was more thermally stable than the unirradiated complexes (Abdalla et al., 2021).

### 3.8 Molecular structure

**FIGURE 2:**
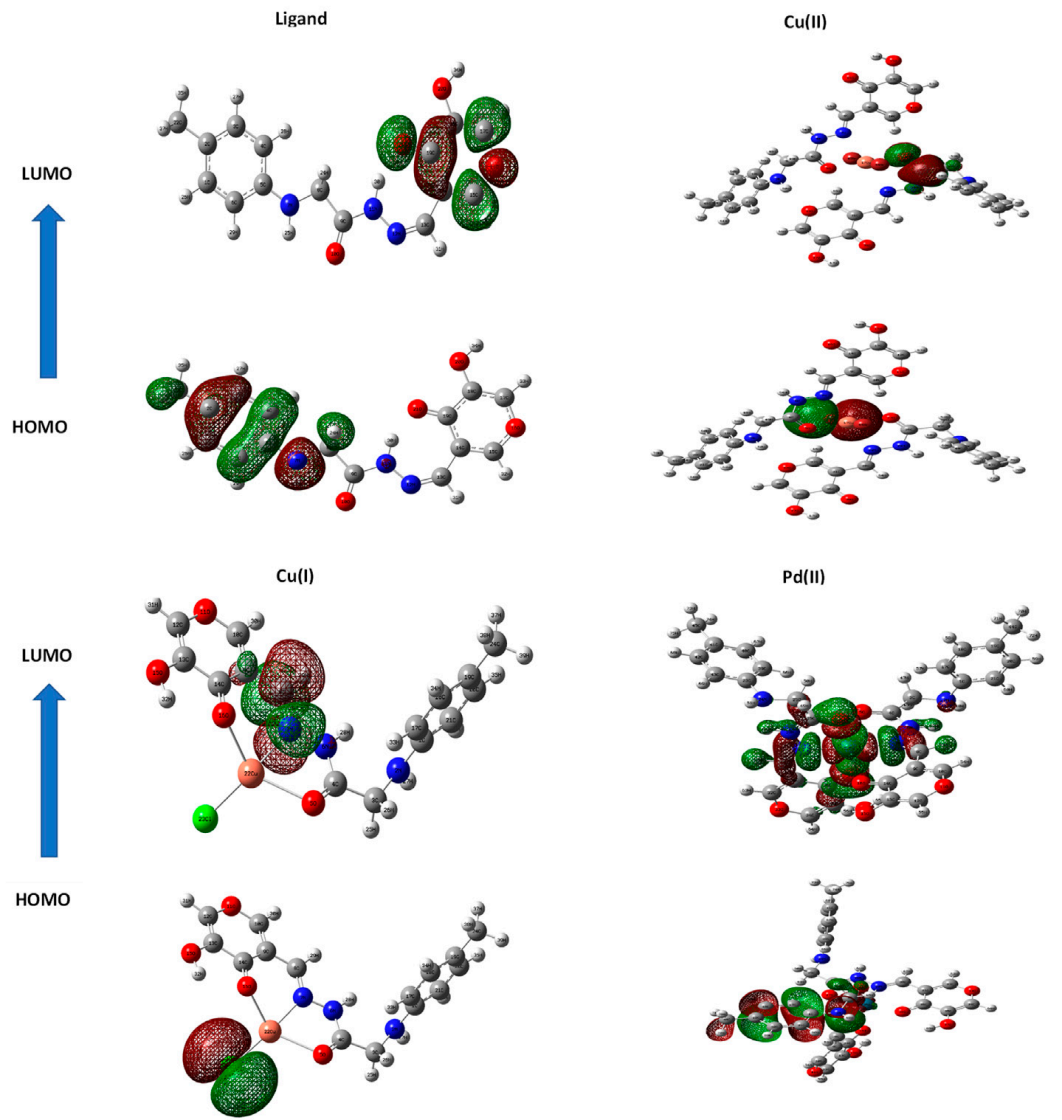
Molecular graphs of all compounds.

When doing quantum chemistry research, the LUMO (p acceptor) and HOMO (p donor) molecular structures of H_2_L_B_ are important considerations. Frontier molecular orbitals (FMOs) are the name given to these molecular orbitals. In HOMO and LUMO (Figure 2), the molecular structures of H_2_L_B_ and its complexes were shown. The energies (E_HOMO_, E_LUMO_) of H_2_L_B_ and its complexes are tabulated in Table 6. Both the E_HOMO_ and E_LUMO_ had negative values, indicating the stability. (ΔE = E_LUMO_ - E_HOMO_) was the energy band gap that related to the charge transfer interface within the LUMO-HOMO of the molecule as specified in Table 6. The equations for dipole moment D), hardness η), softness σ), chemical potential μ), and electronegativity χ) have been obtained and the corresponding results are shown in Table 6.

$$\begin{array}{c} \eta =\left(I-A\right)\;/2\;S=1/\;2\eta \;\mu =-\left(I+A\right)/2\;\chi =\left(I+A\right)/2\eta \\ I=-\;E_{\text{HOMO}}\;A=-\;E_{\text{LUMO}}\; \end{array}$$

Where I = the ionization potential of the molecule.

**TABLE 6:** Ground state criteria of ligand and Cu(I), Cu(II), and Pd(II) complexes using B3LYP/6-311G and B3LYP/LANL2DZ, respectively.

Parameter	H_2_L_B_	Cu(I) complex	Cu(II) complex	Pd(II) complex
E_T_, Hartree	−1,045.39976321	−1,256.76145511	−2,313.75848285	−2,217.43842302
E_HOMO_, eV	−4.64634638	−5.4417356	−6.05834587	−9.87937052
E_LUMO_, eV	−2.41038572	−1.7349988	−1.75186987	−8.78166269
ΔE, eV	2.23596066	3.706737	4.306476	1.097708
I = − E _HOMO_, eV	4.64634638	5.4417356	6.058346	9.87937052
A = − E _LUMO_, eV	2.41038572	1.7349988	1.75187	8.78166269
χ, eV	3.156018004	1.936132727	1.813597879	17.00000009
η, eV	1.11798033	1.853368385	2.153238	0.548853915
S, eV^-1^	0.44723506	0.26977907	0.232208423	0.91098922
µ, eV	−3.52836605	−3.588367185	−3.90510787	−9.330516605
Dipole Moment (Debye)	10.5480	14.6557	2.3535	8.0239

A = electron affinity of the molecule.

Hardness and softness, frequently used as chemical reactivity and stability indicators, were crucial parameters.

The molecule had a narrow gap between the HOMO and LUMO orbitals and became more reactive and softer, as smaller hardness ratings suggested higher reactivity. The order of softness was Pd > Ligand > Cu(I) > Cu(II). Except for the Pd(II) complex, every complex had a greater energy gap than the ligand. Therefore, the stability of most complexes under investigation was higher than the free ligand. The greater reactivity of the Pd II) complex may be explained by the higher polarizability of the second-row transition elements (Palladium) in comparison to the first-row elements (Copper).

**FIGURE 3:**
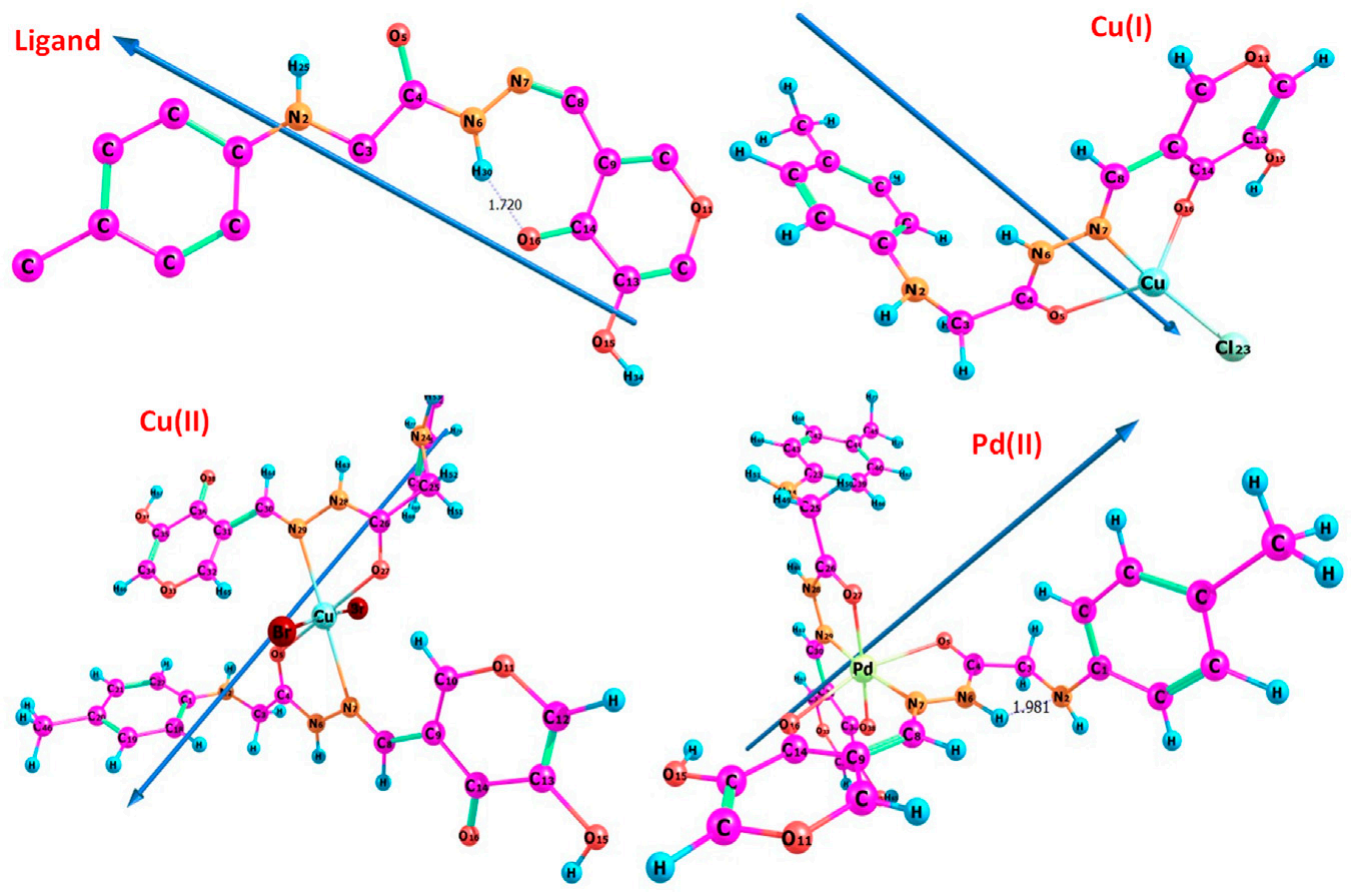
Optimized geometry of all compounds.

The atom ordering of H_2_L_B_ was given in the molecular structure, and complexes were also presented (Figure 3). The following observations were based on the calculated bond lengths and orientations directed at the compounds (Table 7).

**TABLE 7:** Several bond lengths, Å and bond angles, degrees, for H_2_L and its metal chelates.

Bond length (A^o^)	H_2_L_B_	Cu(I) complex	Cu(II) complex	Pd(II) complex
R(C3-C4)	1.52898	1.53384	1.54370	1.53908
R(C4-O5)	1.24188	1.25226	1.26037	1.25313
R(C4-N6)	1.38249	1.38460	1.36856	1.38569
R(N6-N7)	1.36156	1.38528	1.39872	1.39448
R(N7-C8)	1.29829	1.31158	1.30142	1.31484
R(C8-C9)	1.48124	1.46039	1.46045	1.45667
R(C9-C14)	1.47007	1.46633	1.46249	1.46384
R(C14-O16)	1.26148	1.27469	1.27521	1.27649
R(H-N6)	1.03165	1.02151	1.01990	1.03504
R(O16-M)	---	2.26158	---	2.61590
R(N7-M)	---	2.06972	2.54149	2.07748
R(O5-M)	---	2.42471	2.03551	2.69381
R(X-M)	---	2.22424	2.53713	
Bond angles, degrees				
A(O16-C14-C9)	124.823	125.850	125.321	126.073
A(C14-C9-C8)	127.301	122.699	116.356	127.037
A(C9-C8-N7)	136.152	122.535	124.071	130.569
A(C8-N7-N6)	123.820	117.748	115.942	115.769
A(N7-N6-C4)	119.717	118.628	120.555	125.434
A(N6-C4-O5)	125.039	122.475	122.740	124.934
A(N6-C4-C3)	112.721	114.679	114.841	111.429
A(C4-C3-N2)	108.710	114.081	115.926	112.370
A(H-N6-N7)	118.934	123.115	119.567	121.790
A(O16-M-N7)	---	82.054	---	83.561
A(O5-M-N7)	---	74.188	73.428	74.038
A(C14-O16-M)	---	116.166	---	119.855
A(C8-N7-M)	---	127.184	143.087	129.815
A(Br-Cu-Br)	---	---	176.376	---
A(Cl-Cu-O5)	---	116.443	---	---
A(Cl-Cu-O16)	---	108.623	---	---

In the complexes some bond lengths were increased [(C3-C4), (N7-C8), (C14-O16), (N6-N7) and (C4-O5)] and others were decreased [(C8-C9) and (C9-C14)] to adjust the coordination via the N7,O5, and O16-atoms in both Cu(I) and Pd(II) complexes with the emergence of new O16-M, (N7-M), and (O5-M) bonds. In the case of the Cu (II) complex, the coordination was carried out through N7 and O5 atoms comp, leaving the coordination sphere via bromide bonding. As seen in Table 7, coordination modified the H_2_L_B_ bond angles, and when the metal center was changed, significant changes in the angles surrounding the metal also occurred. The significant swing in angle values due to bonding during complex formation was either increased or decreased. N7, O5, and O16 atoms in the ligand calculated natural charges of −0.197, −0.583, and −0.589 respectively as seen in Supplementary Table S1. Charge transfer from L(ligand)→M(metal) could be supported by decreased calculated charges on metal ions after coordination (Hassan and Gomha, 2019). The charges changed from Cu (I), Cu (II), and Pd (II) to 0.503, 0.0255, and 0.488 respectively. The theoretical results of FTIR showed good agreement with the experimental results as seen in Table 2 and Supplementary Figures S3-S6. The theoretical infrared spectra of the ligand L showed noteworthy absorption regions at 344, 1700, 1,633, and 1,597 cm^-1^ corresponding to v N–H), (C=O)side, (C=O)ring, and (C=N) vibrations. However, when these vibrations were complexed with metal ions, their positions shifted from their initial values. Also, the electronic transitions of ligands and complexes in the presence of solvent effect were calculated and the transition values are tabulated in Table 3.

### 3.9 Antimicrobial study

The antibacterial properties of the H_2_L and its chelates were tested against different Gram-positive and negative strains of bacteria both before and after exposure to gamma irradiation. Additionally, it had antifungal effects against *Candida albicans* and *Aspergillus Nigar*. The conventional antimicrobial agents used for antibacterial and antifungal research purposes included ampicillin, gentamicin, and nystatin.

**FIGURE 4:**
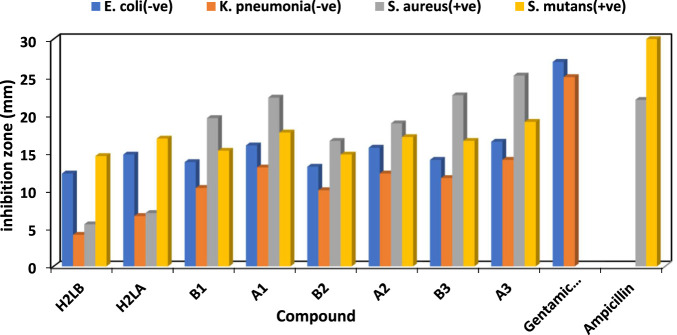
Antibacterial results (*In vitro*) of H_2_L and chelates before (B) and after (A) irradiation

**FIGURE 5:**
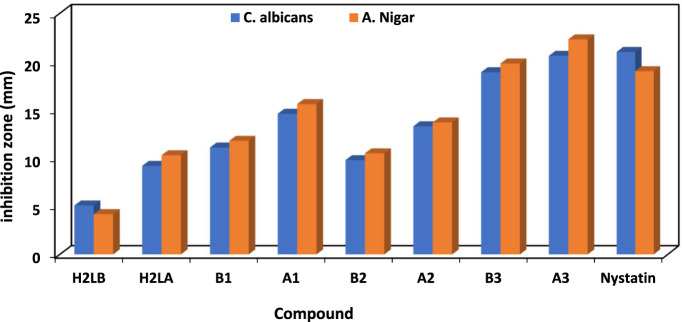
*In vitro* antifungal results of H_2_L and its chelates before (B) and after (A) irradiation.

The efficacy of the antibacterial properties of all produced compounds is demonstrated in Supplementary Table S2 and Figure 4 and Figure 5. The findings suggested that complexes exhibited more activity compared to ligands. Furthermore, following irradiation, complexes demonstrated enhanced efficacy as antibacterial and antifungal agents, surpassing their pre-irradiation performance (Aly and Elembaby, 2020a; Abdalla et al., 2021). The increased activity of complexes may be elucidated by Overtone’s notion (Anjaneyulu and Rao, 1986), as evidenced in our prior studies (Khalf-Alla et al., 2019; Hassan and Khalf-Alla, 2020; Hassan and Mohamed, 2020). The reduction in polarity of the metal ion occurred as a result of chelation, due to the interaction between its partial positive charge and the donation sites of the coordinated ligand. Additionally, chelation promoted the dispersion of π-electrons throughout the whole chelate ring, hence enhancing the lipophilic nature of the chemical under investigation. The compound’s lipophilic nature facilitated its penetration through the lipid layer of the cell membrane, resulting in a more potent and destructive effect on the cells. Moreover, the Cu(I) complex exhibited enhanced antibacterial activity after irradiation (A_3_), surpassing the antibacterial activity of the Cu(I) complex before irradiation (B_3_) against bacterial species compared to other substances, including standard drugs like ampicillin and gentamicin. Similarly, when nystatin was used as a standard drug, the Cu(I) complex also displayed superior antifungal activity.

### 3.10 Evaluation of docking studies

A nitrogen-containing heterocyclic molecule is an essential scaffold with antibacterial potential. Therefore, our research aimed to synthesize and investigate new nitrogen-containing heterocyclic compounds experimentally *in vitro* against different bacterial strains and theoretically using MOE 2008 software (Hassan and Mohamed, 2020). A molecular docking study was performed with the target site of topoisomerase enzyme(2xct) chain a from the protein data bank. The topoisomerase enzyme is a crucial enzyme that plays a vital role in the process of DNA replication (Redgrave et al., 2014). The gyrase enzyme facilitates the relaxing of super-coiled DNA during DNA replication by breaking and rejoining both strands of the DNA chain, allowing for unwinding and replication (Champoux, 2001).

**FIGURE 6:**
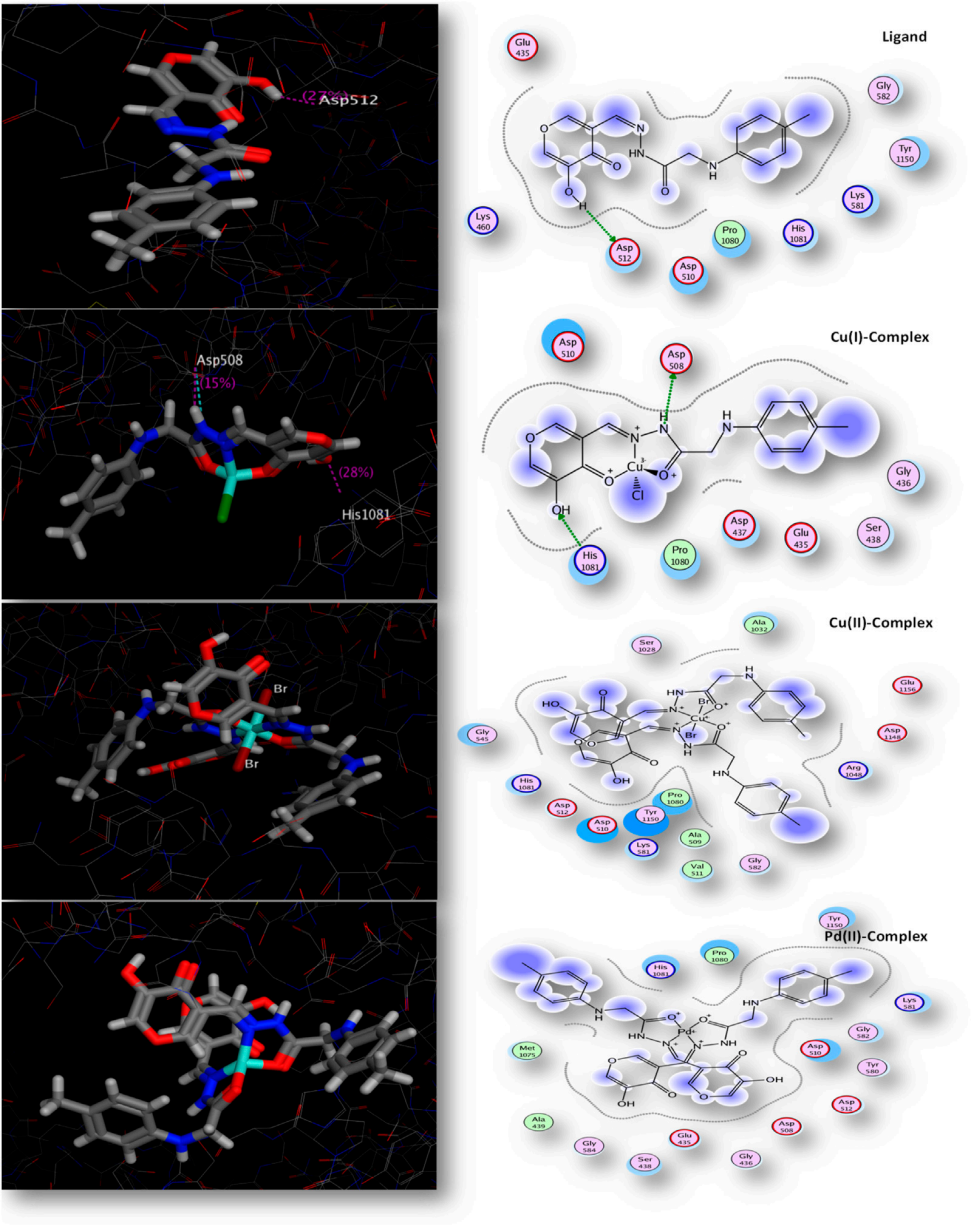
2D and 3D interaction ability of all compounds with topoisomerase IIa enzyme (code: 2xct).

There was a good correlation between the *in vitro* antimicrobial inhibition assay and the scoring energy values, as shown in Table 8 and Supplementary Table S3. The Palladium (II) and Copper (1) complexes revealed effective results against most microbes. The explored binding affinity was the best-posed interaction with low root mean square deviation values (RMSD). Figure 6 displays the different binding interaction types of compounds with 2xct protein. The Copper(I) complex showed sidechain acceptor and sidechain donor interaction types with Asp-508 and His1081 amino acid residues, respectively. The reactivity sequence relative to the scoring energy values was Cu(I) > Pd(II) > Cu(II) > L, which showed good fitting with the zone inhibition values experimentally where the Cu(I) complex inhibited bacterial growth in both Gram-positive and Gram-negative bacteria, with zone inhibition values that were greater than those of the Pd(II), Cu(II), and ligand compounds. The binding affinity of our compounds consistently demonstrated superior or equivalent values in multiple prior studies targeting the same protein type (Pisano et al., 2019; El-Etrawy and Sherbiny, 2021). Paraphrased, (Several compounds were examined before against the same 2xct protein and observed high affinity to inhibit the examined protein with scoring energies − 9.41 relative to Cu(II) chelate) (Aly et al., 2023). Our new Cu(I) chelate observed a similar affinity to the 2xct protein in the present paper. Also, our compounds achieved a 2xct protein inhibition effect in the docking scoring range (−3.63 to −8.51) observed for the quinolone moiety-based compounds (Patel and Patel, 2014).

**TABLE 8:** Topoisomerase IIa enzyme (code: 2xct) interaction with all compounds.

Docking 2xct			
Compound	Scoring energy (RMSD)	Active amino acids	Interaction type
H_2_L_B_	−3.7278(1.22)	Asp-512	Sidechain acceptor
Cu(I)	−7.2839(2.39)	Asp-508 and His1081	Side chain acceptor and Sidechain donor
Cu(II)	−4.1185(2.34)	---	Solvent contact
Pd(II)	−6.6027(2.34)	---	Solvent contact

## 4 Conclusion

Based on a novel (H_2_L_B_) ligand (Z)-2-(phenylamino)-N'-(thiophen-2-ylmethylene) acetohydrazide), three new chelates, Pd(II), Cu(II), and Cu(I), have been isolated. Based on the outcomes of various characterization approaches, the hypothesized structures of the (H_2_L_B_) and its chelates verified the production of 1:2 Pd(II) and Cu(II) and 1:1 (M:L) Cu(I) chelates. The molar conductance values of the chelates demonstrated their non-electrolytic character except for the ionic Pd(II) Complex. The effectiveness of the antibacterial and antifungal treatments was compared to the industry-standard medications ampicillin, gentamicin, and nystatin. Paraphrased, (Zone inhibition values revealed that the Cu(I) complex after irradiation (A3) acquired better antibacterial activity followed by the Cu(I) complex before irradiation (B3) relative to bacterial species than others, when ampicillin and gentamicin as reference drugs, as well as the antifungal species when nystatin was used as a standard drug). The antimicrobial activity of these complexes followed the order: Cu(I) complex > Pd(II) complex > Cu(II) complex > Ligand. The geometries of the Pd(II) complex were square planar, according to the DFT calculations performed on the synthesized compounds. Cu(II) was octahedral, with hex coordinates chosen around the metal ions, while Cu(I) complex had a tetrahedral geometry. The synthesized ligand and the range of antibacterial activity of all the metal complexes against bacterial species were satisfactory. The docking stimulation revealed all of the chemicals' binding models.

## Data Availability

The original contributions presented in the study are included in the article/Supplementary Material, further inquiries can be directed to the corresponding authors.
