# Supplementary material for: Synthesis, characterization, PXRD studies, and theoretical calculation of the effect of gamma irradiation and antimicrobial studies on novel Pd(II), Cu(II), and Cu(I) complexes
Source: Front Chem. 2024 Feb 12;12:1357330. doi: 10.3389/fchem.2024.1357330 (PMC10894937; doi:10.3389/fchem.2024.1357330)
Supplement: Supplementary file 1 [file DataSheet1.docx]

**Synthesis, Characterization, PXRD Studies,Theoretical Calculation, the effect of Gamma Irradiation and Antimicrobial Studies on novel Pd(II), Cu(II), and Cu(I) Complexes**

**Section S1: *Physical measurements***

The Fourier Transform Infrared was measured (4000– 400 cm-1) in KBr discs using Nenexeus-Nicolidite-640-MSA FT-IR, Thermo-Electronics Co. In DMF solution the UV–visible absorption spectra were measured by using a 4802 UV/vis double beam spectrophotometer. The ^1^H-NMR spectra have been recorded in DMSO-d_6_ as a solvent using Varian Gemini 200 NMR spectrophotometer and Varian-Oxford Mercury at 300 MHz, respectively. Mass spectra were acquired using the electron impact (EI) ionization technique at 70 eV using a Hewlett–Packard MS-5988 GC–MS instrument at the Microanalytical Center, National Research Centre, Dokki, Cairo, Egypt. Thermal analysis (TG/DTG) was obtained by using a Shimadzu DTA/TG-50 Thermal Analyzer with a heating rate of 10 °C/min in a nitrogen atmosphere with the following rate of 20 ml/min using platinum crucibles in the range of ambient temperature up to 800 °C. X-ray powder diffraction analyses of solid samples were measured using an APD 2000 PROModel GNR-X-ray diffractometer at (NRC, Tanta University, Egypt). X-ray diffractograms give computer control formally finished by PHILIPS®MPDX´PERT X-ray diffractometer ready with Cu radiation CuKα (λ=1.540 56 Å). Most powder diffractometers use Bragg-Brentano parafocusing geometry. The x-ray tube applied was a copper tube operating at 40 KV and 30 mA. The scanning range (2θ) was 5–90° with a step size of 0.050° and a counting time of 2 s/step. Quartz was utilized as the standard material to be accurate for the instrumental extension. This identification of the complexes was done by a known method from the fit identified Scherer formula, the average crystallite size (D) is: D = (Kλ /β cos θ) Where: λ is the X-ray wavelength in the nanometer, K is a factor related to crystallite shape, and with a value of about 0.9 and ß is the peak width at half maximum height. The value of ß in the 2θ pivot of diffraction shape should be in radians. The θ is the Bragg angle and is able to be in radians since Cos θ is suitable with the same number.

**Section S2. Antimicrobial screening studies**

In the antimicrobial screening, each of the compounds was dissolved in DMSO, and a solution of the concentration 1 mg/mL was prepared separately; paper disks of Whatman filter paper were prepared with standard size (6 mm), cut and sterilized in an autoclave. The paper disks, soaked in the desired concentration of the complex solution, were placed aseptically in the Petri dishes containing nutrient agar media (agar 20 g + beef extract 3 g + peptone5g) seeded with *Staphylococcus aureus, Streptococcus mutants, E. coli, Klebsiella* *pneumonia, Candida albicans, and Asperagillus Nigar.* The Petri dishes were incubated at 36 °C, and the inhibition zones were recorded after 24 h of incubation. Each treatment was replicated three times. The antibacterial activity of common standard drugs *for antibacterial and fungal research included Ampicillin, Gentamicin and Nystatin.*

**Section S3.Irradiation studies**

All samples were taken in a dose of 60 kGy using an Indian ^60^Co γ-ray cell type GE-4000 A (at room temperature at the Egyptian Atomic Energy Authority Nasr City, Egypt) at a dose rate of 2.2 kGy h^-1^. After removing the samples from the radiation field the FT-IR, absorption spectra, thermal analysis (TG/DTG), PXRD, and antimicrobial activity were investigated by the same methods used before for the irradiated compound.

**Table S1** Atomic charges in terms of MPA and NPA of all synthesized compounds

| **Element** | **NPA(MPA)** | | | |
| --- | --- | --- | --- | --- |
|  | H_2_L | Cu(I) complex | Cu(II) complex | Pd(II) complex |
| **M** | --- | 0.503(0.187) | 0.0255(-0.056) | 0.725(0.487) |
| **N2** | -0.604 (-0.815) | -0.717 (-0.467) | -0.335(-0.409) | -0.737(-0.501) |
| **C3** | -0.269 (-0.268) | -0.283 (-0.472) | -0.138(-0.517) | -0.278(-0.462) |
| **C4** | 0.665 (0.559) | 0.713 (0.336) | 0.375(0.513) | 0.715(0.325) |
| **O5** | -0.583 (-0.382) | -0.600 (-0.262) | -0.344(-0.300) | -0.621(-0.279) |
| **N6** | -0.436 (-0.588) | -0.456 (-0.373) | -0.222(-0.385) | -0.466(-0.388) |
| **N7** | -0.197 (-0.076) | -0.319 (-0.050) | -0.130(0.111) | -0.261(-0.053) |
| **C8** | 0.014 (-0.050) | 0.086 (-0.270) | 0.056(-0.321) | 0.147(-0.213) |
| **C9** | -0.214 (-0.251) | -0.199 (0.322) | -0.111(0.281) | -0.223(0.326) |
| **C14** | 0.424 (-0.138) | 0.431 (0.154) | 0.214(0.061) | 0.435(0.144) |
| **O15** | -0.633(-0.559) | -0.703(-0.443) | -0.355(-0.452) | -0.683(-0.415) |
| **O16** | -0.589 (-0.428) | -0.619 (-0.324) | -0.328(-0.335) | -0.643(-0.341) |
| **Br** | --- | --- | -0.345(-0.385) | --- |
| **Cl** | --- | -0.603(-0.415) | --- | --- |

**Table S2:** Results of antimicrobials bioassay of ligand (H_2_L) and its complexes of Pd(II), Cu(II), and Cu(I) before and after gamma irradiation against different strains of bacteria and fungi

| Sample  Microorganism | H_2_L_B_ | H_2_L_A_ | Pd(II) complex  B_1_ | Pd(II) complex  A_1_ | Cu(II) complex  B_2_ | Cu(II) complex  A_2_ | Cu(I) complex  B_3_ | Cu(I) complex  A_3_ | Standard antibiotic |
| --- | --- | --- | --- | --- | --- | --- | --- | --- | --- |
| **Gram negative bacteria** |  | | | | | | | | Gentamicin |
| *Escherichia coli*  *(ATCC:10536)* | 12.3±0.5 | 14.8±0.5 | 13.8±0.5 | 16.0±0.5 | 13.2±0.5 | 15.7±0.5 | 14.1±0.5 | 16.5±0.5 | 27±0.5 |
| *Klebsiella pneumonia*  *(ATCC:10031)* | 4.2±0.6 | 6.7±0.6 | 10.4±0.6 | 13.1±0.6 | 10.1±0.5 | 12.3±0.6 | 11.7± 0.5 | 14.1± 0.5 | 25±0.5 |
| **Gram positive bacteria** |  | | | | | | | | Ampicillin |
| *Staphylococcus aureus*  *(ATCC:13565)* | 5.6±0.5 | 7.1±0.5 | 19.6±0.6 | 22.3±0.5 | 16.6±0.6 | 18.9±0.6 | 22.6±0.6 | 25.2±0.6 | 22±0.1 |
| *Streptococcus mutans*  *(ATCC:25175)* | 14.6±0.5 | 16.9±0.5 | 15.3±0.5 | 17.7±0.5 | 14.8±0.5 | 17.1±0.5 | 16.6±0.5 | 19.1±0.5 | 30±0.5 |
| **Fungi** |  | | | | | | | | Nystatin |
| *Candida albicans*  *(ATCC:10231)* | 5.1±0.6 | 9.2±0.6 | 11.1±0.5 | 14.6±0.6 | 9.8±0.6 | 13.3±0.5 | 18.9±0.6 | 20.6±0.5 | 21±0.5 |
| *Asperagillus Nigar*  *(ATCC:16404)* | 4.2±0.6 | 10.3±0.5 | 11.8±0.5 | 15.6±0.6 | 10.5±0.6 | 13.7±0.6 | 19.8±0.6 | 22.3±0.6 | 19±0.5 |

**Table S3**: output results of docking investigation

| **Compound** | **S** | **Rmsd_refine** | **E_conf** | **E_place** | **E_refine** |
| --- | --- | --- | --- | --- | --- |
| **H_2_L_B_** | -3.7278 | 1.2225 | 93.9599 | -66.3076 | -16.8751 |
| **Cu(I)** | -7.2839 | 2.3907 | 55.5334 | -71.0217 | -22.4234 |
| **Cu(II)** | -4.1185 | 2.3425 | -93.1670 | -63.2584 | -20.5755 |
| **Pd(II)** | -6.6027 | 2.3435 | 102.4103 | -104.5907 | -14.5730 |


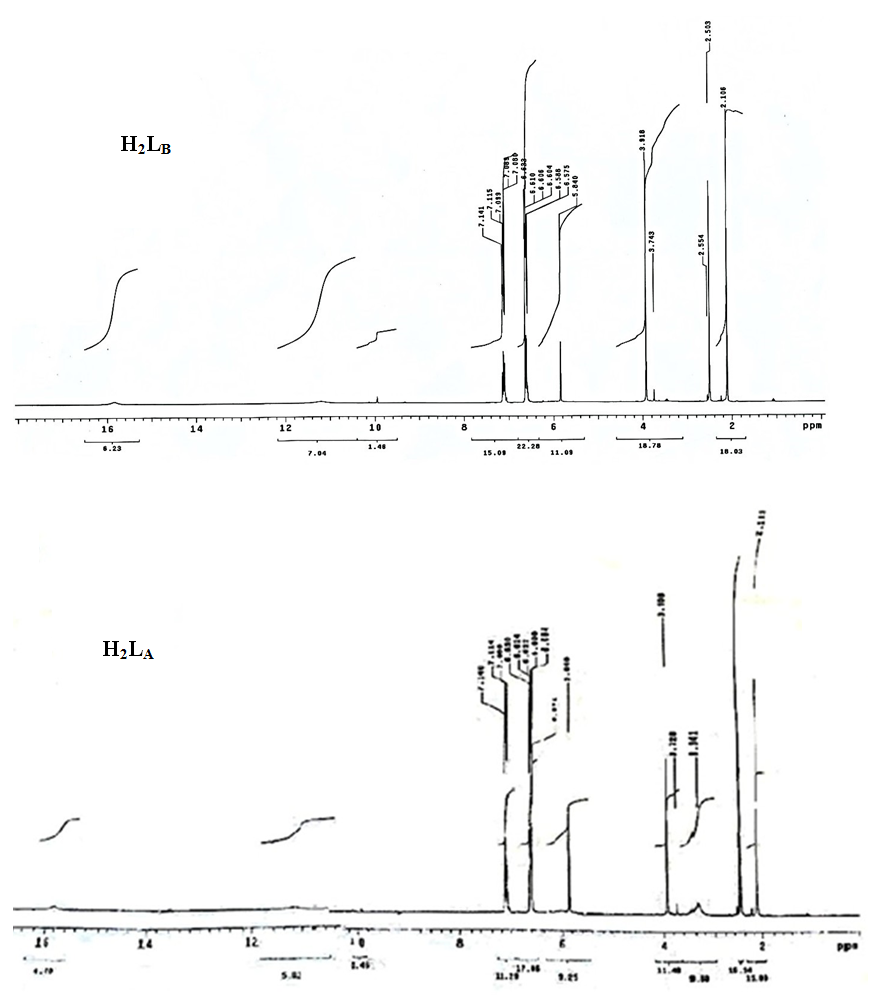


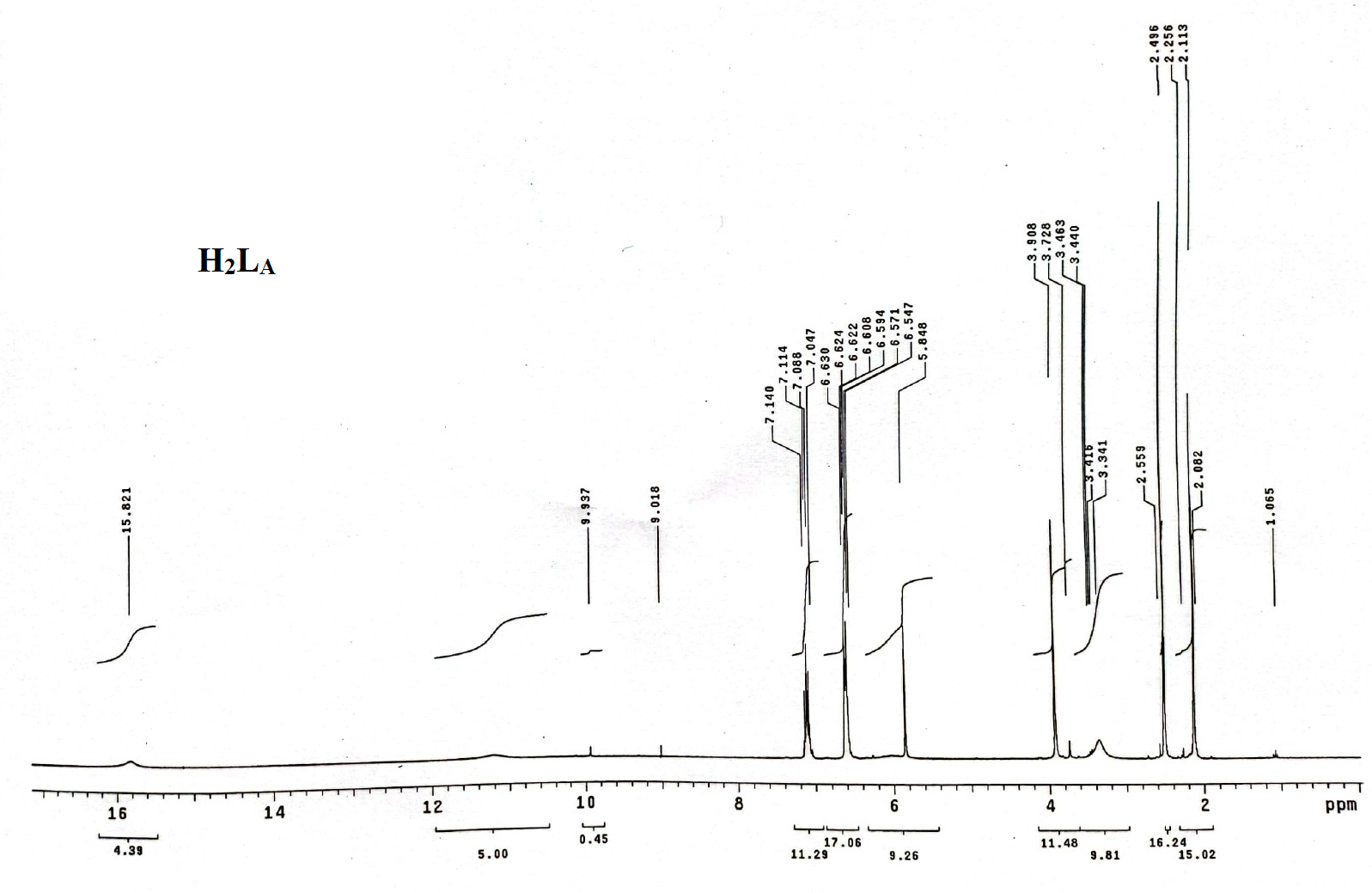


**Figure S1** ^1^H-NMR of ligand before H_2_L_B_ and after H_2_L_A_ irradiation

***
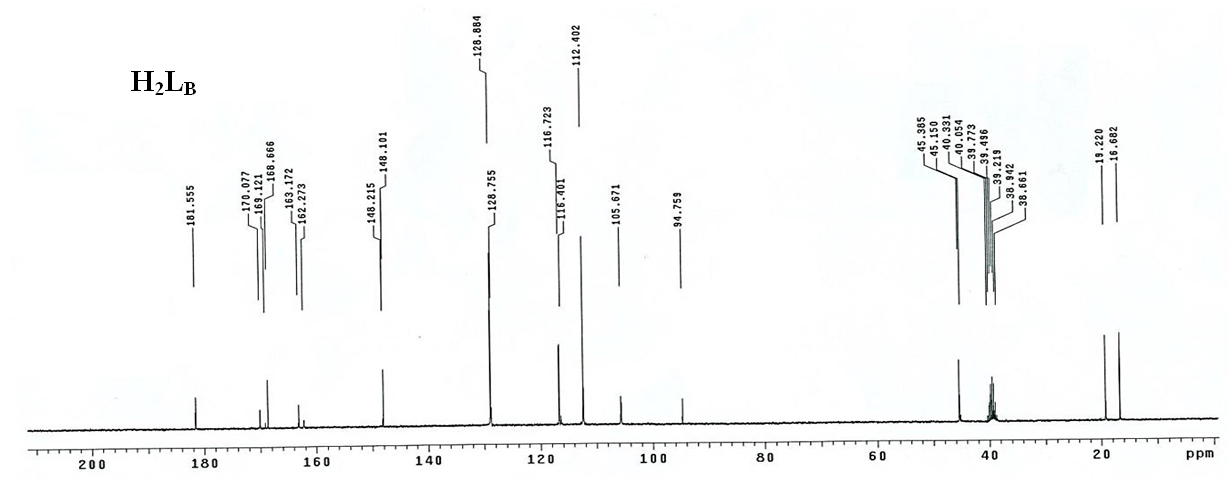
***

**Figure S2:** ^13^C-NMR spectrum of the ligand (H_2_L_B_) in DMSO _d6_


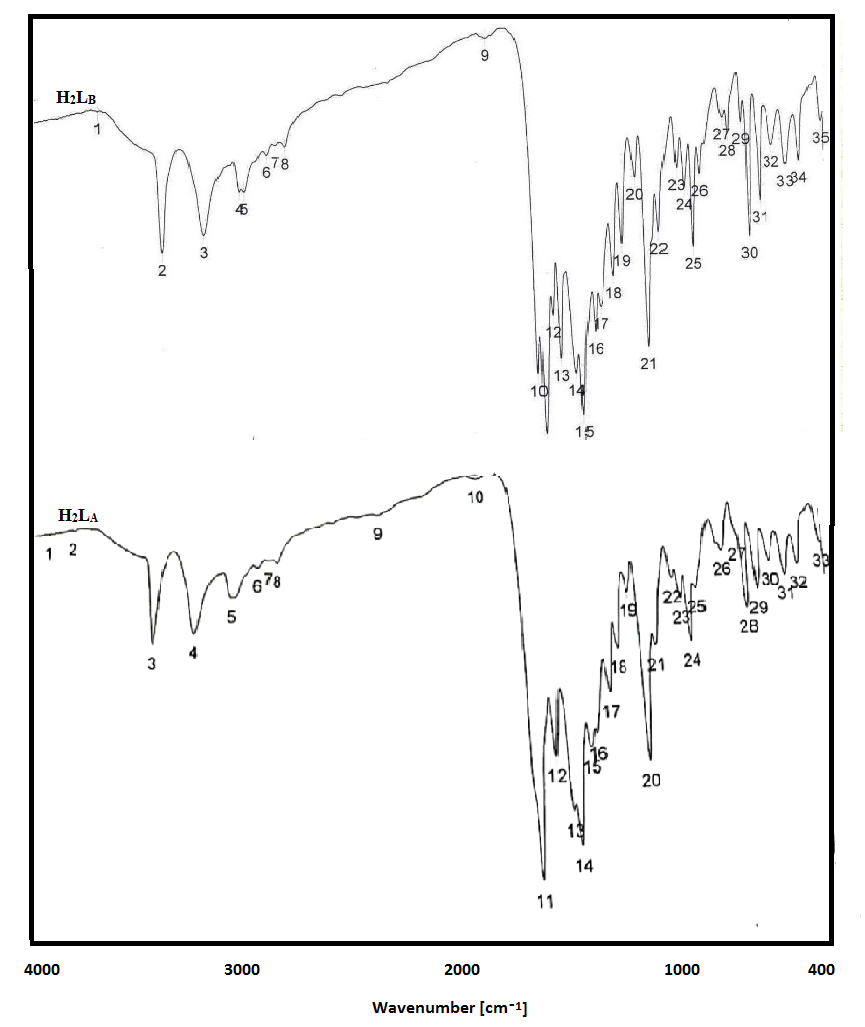


**Figure S3:** FT-IR spectra of ligand before and after irradiation (H_2_L_B_ ,H_2_L_A_).


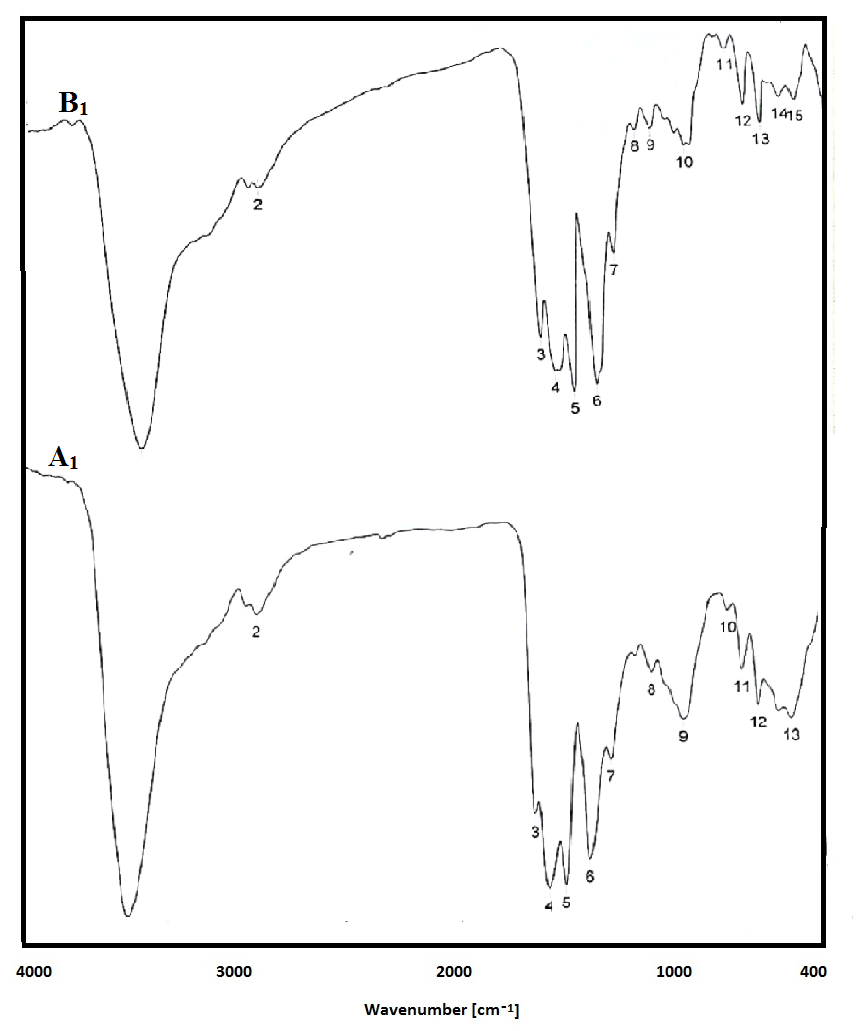


**Figure S4:** FT-IR spectra of Pd(II) chelates (A_1_, B_1,_) before and after irradiation


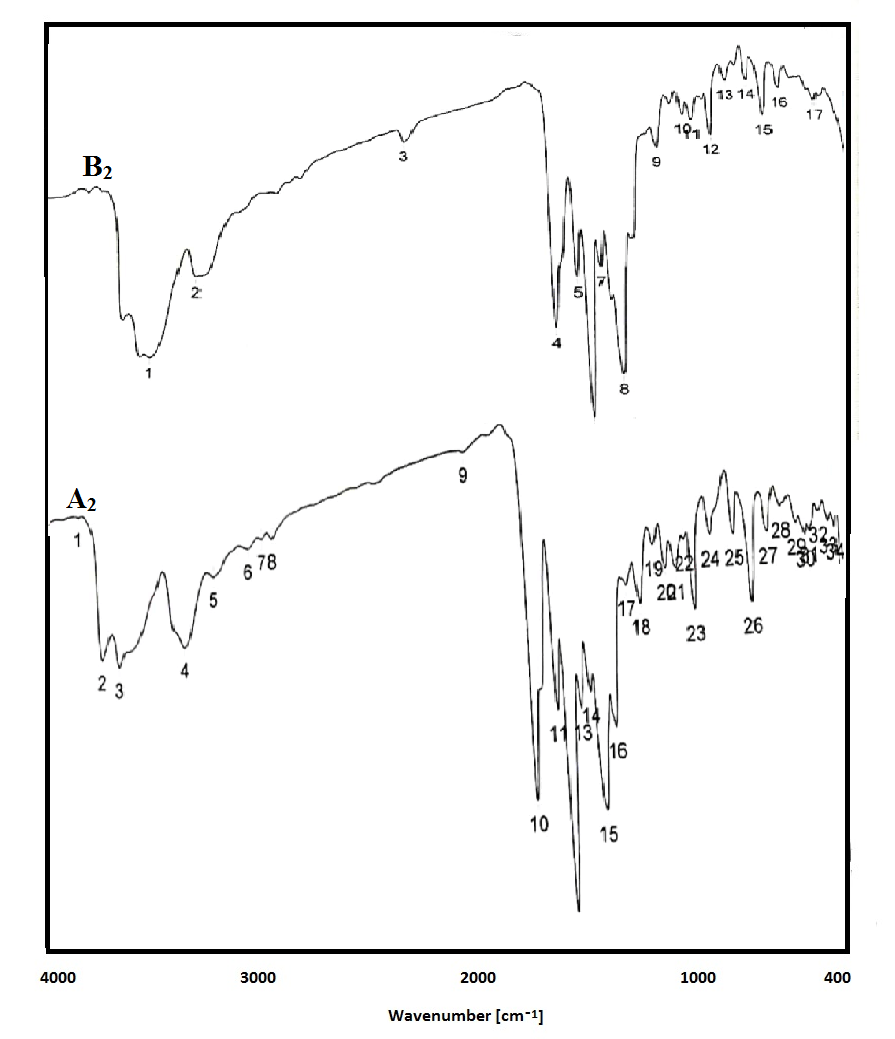


**Figure S5:** FT-IR spectra of Cu(II) chelates (B_2_, A_2_) before and after irradiation


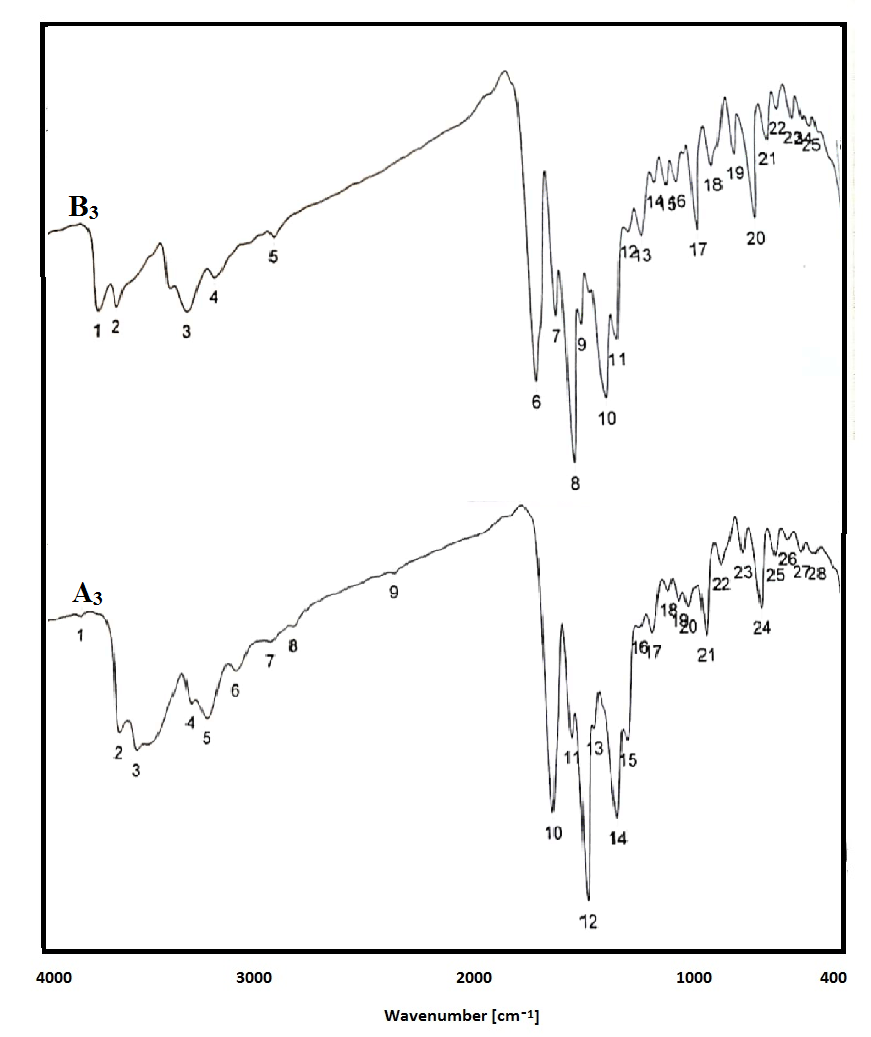


**Figure S6:** FT-IR spectra of Cu(I) chelates (B_3_, A_3_) before and after irradiation.

**Figure S7**: Theoretical FT-IR spectra powder pattern of ligand and its complexes

**
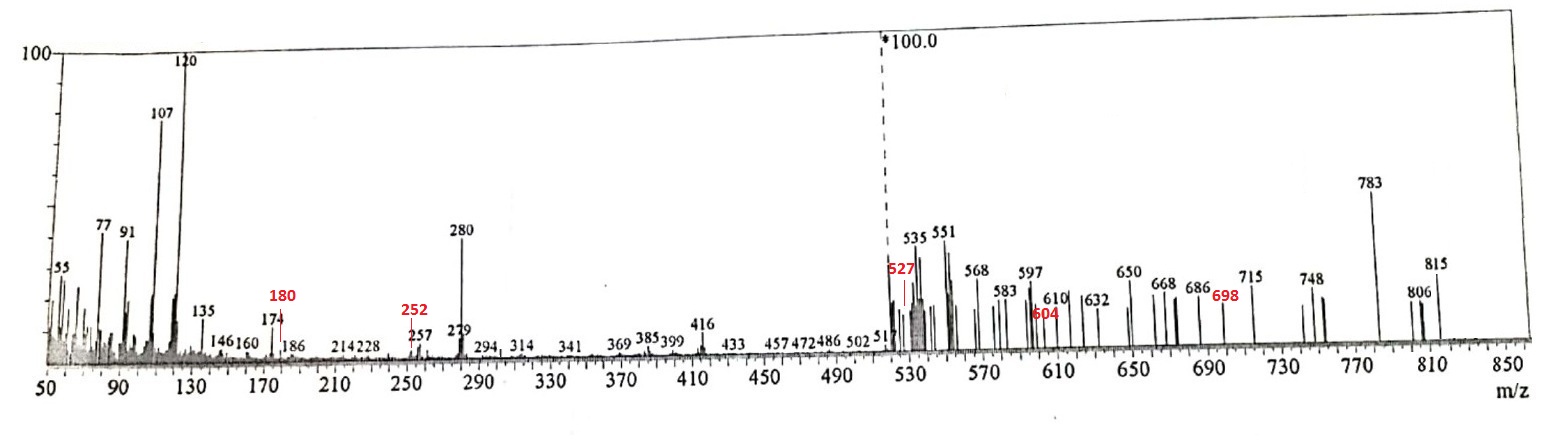
**

**Figure S8:** Mass spectra of the Pd(H_2_L)_2_]Cl_2_.2H_2_O complexes

**
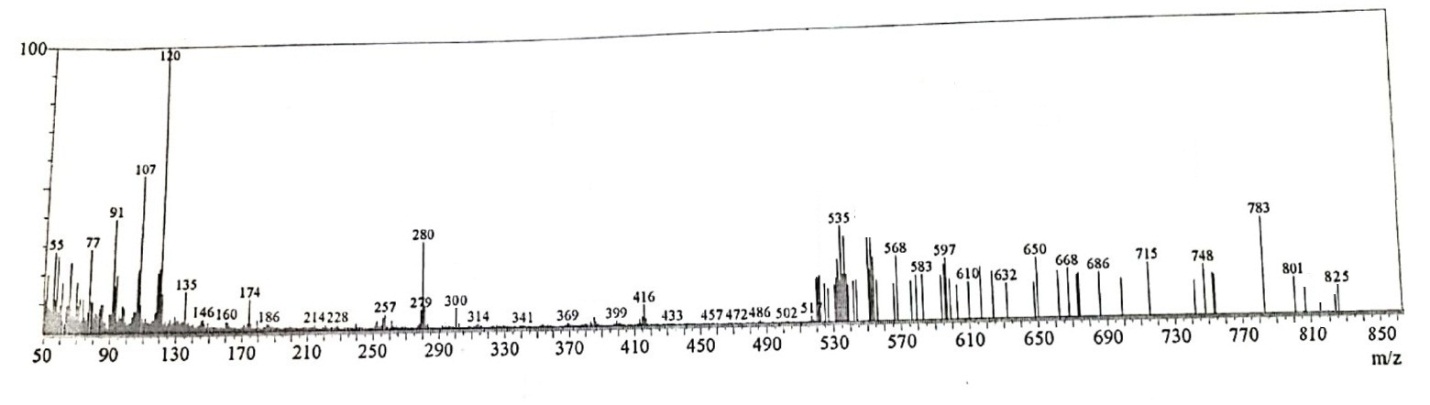
**

**Figure S9:** Mass spectra of the Cu(H_2_L)_2_Br_2_ complexes

**
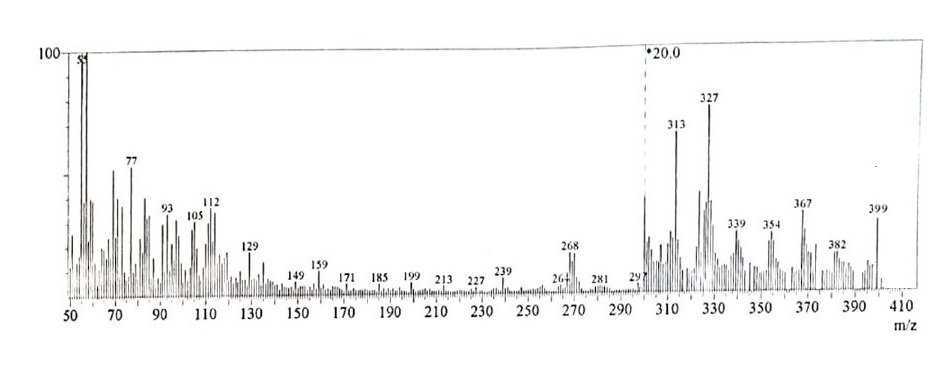
**

**Figure S10:** Mass spectra of the Cu(H_2_L)Cl complexes

**
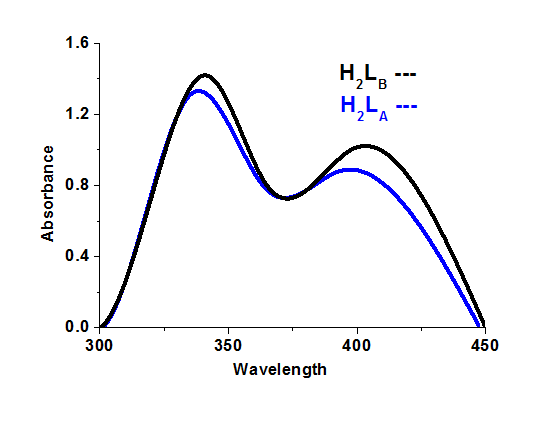

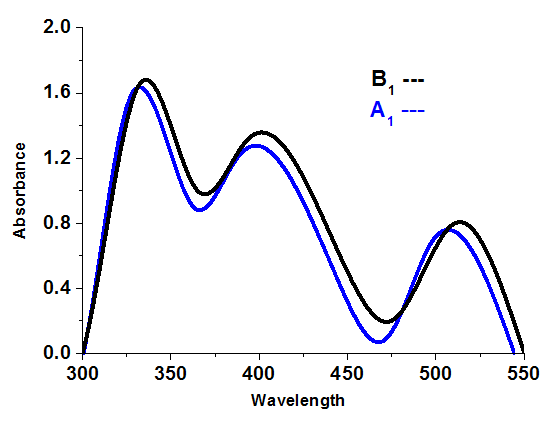
**

**Figure S11**: Electronic spectra of the synthesized of ligand, Pd(II) Complexes before (H_2_L_B_, B_1_,) and after (H_2_L_A_, A_1_) irradiation

**
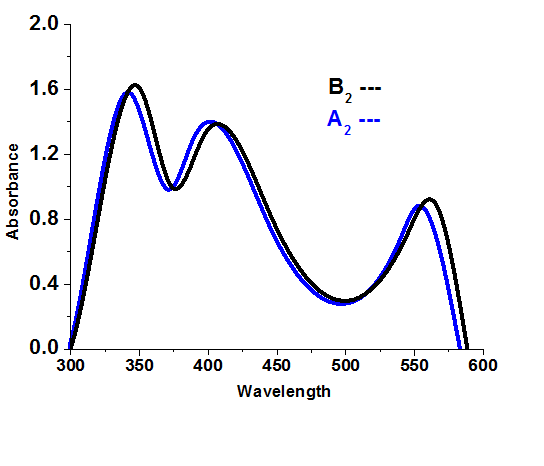

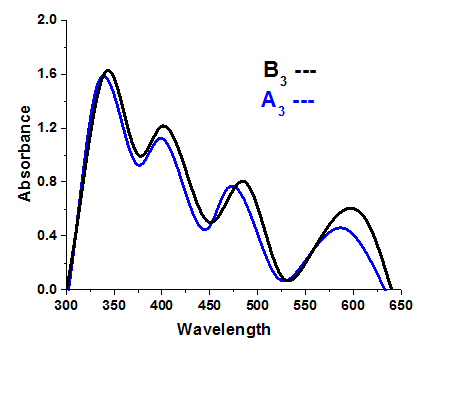
**

**Figure S12**: Electronic spectra of Cu(II), and Cu(I) Complexes before (B_2_, B_3_) and after (A_2_, A_3_) irradiation

**Figure S13**: PXRD powder pattern of ligand before (H_2_L_B_) and after (H_2_L_A_) irradiation


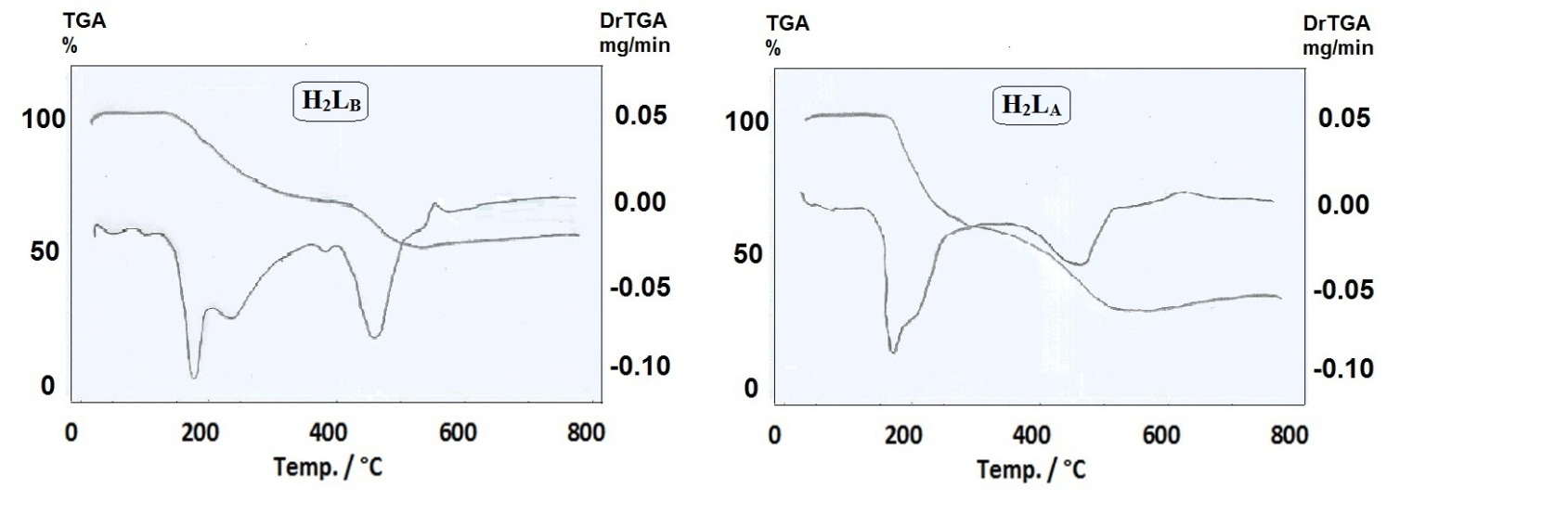


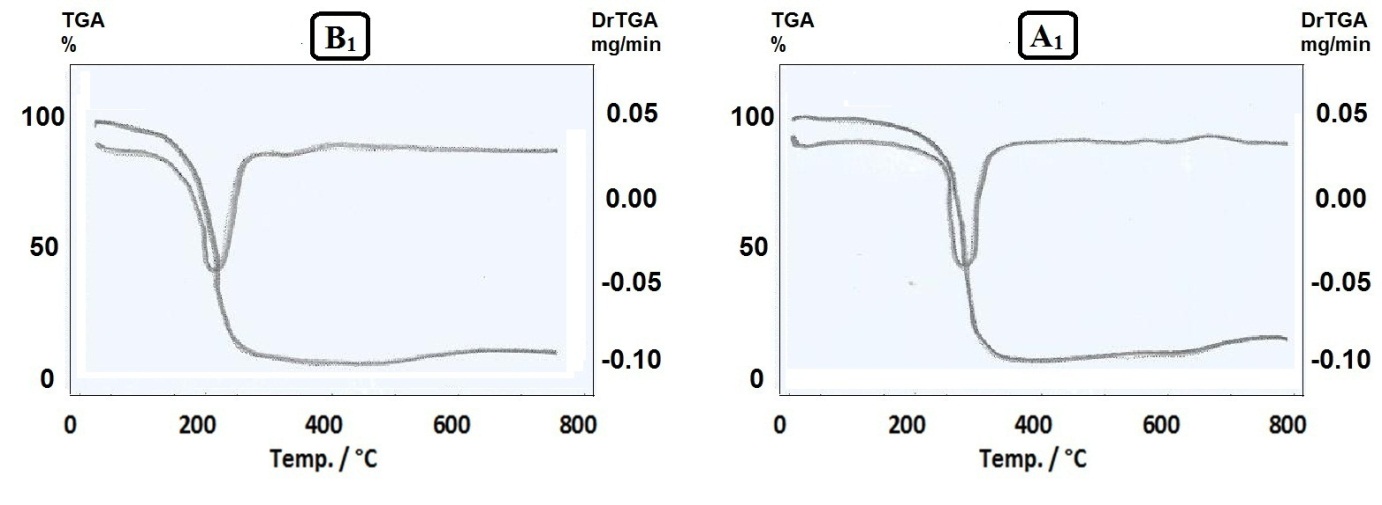


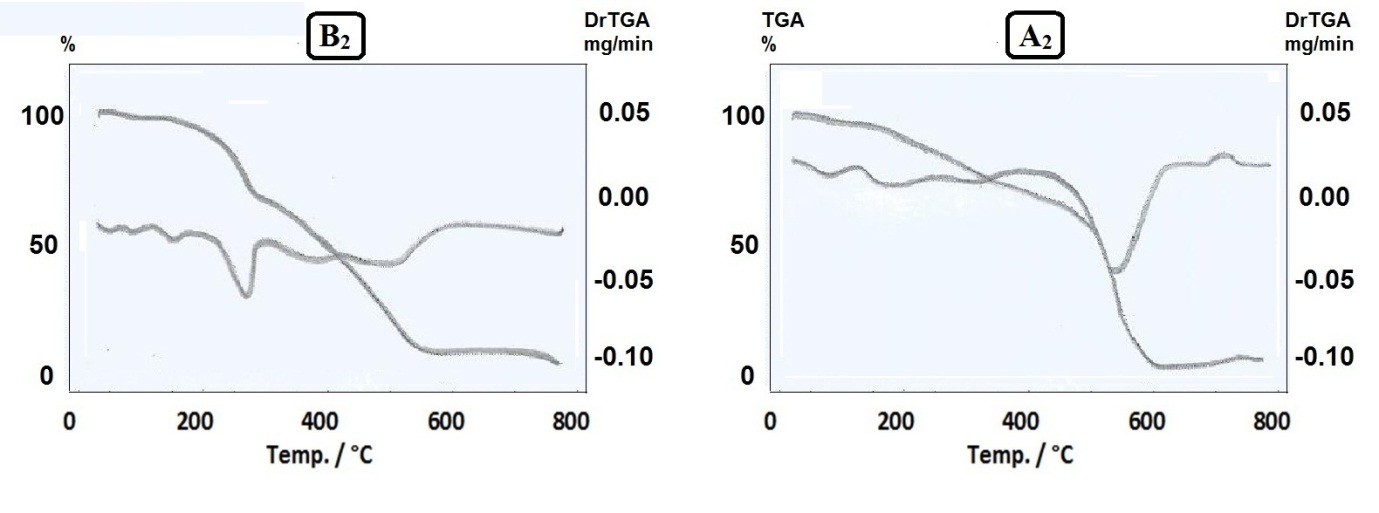


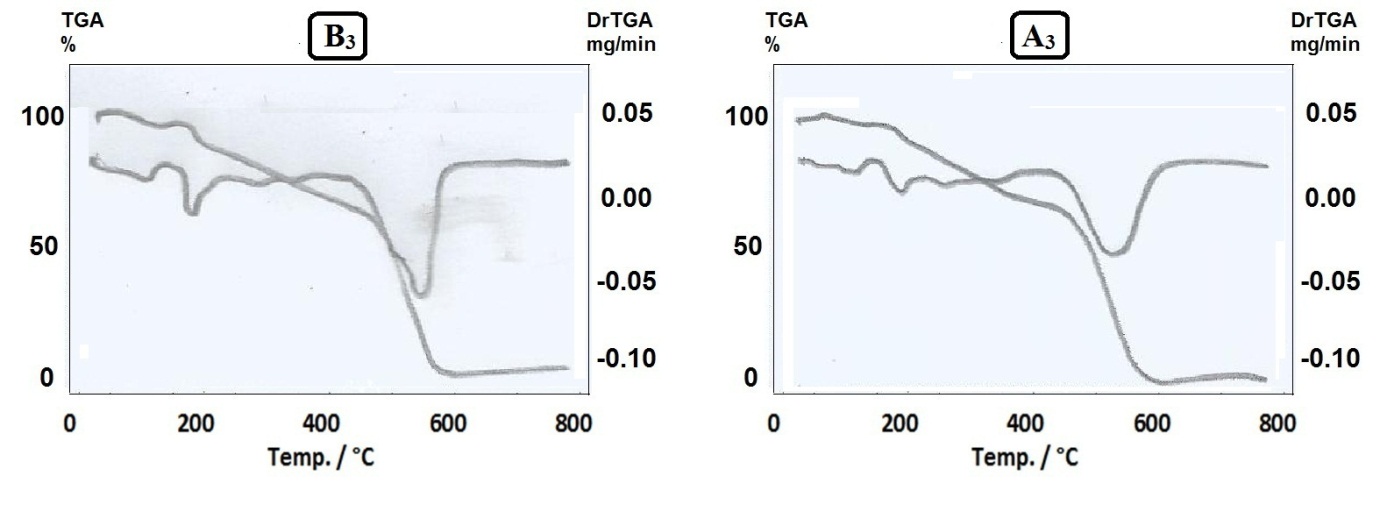


**Figure S14:**  TGA/ DTG curves of the synthesized ligand and complexes before (H_2_L_B,_ B_1,_ B_2_, and B_3_) and after (H_2_L_A,_ A_1,_ A_2,_ and A_3_) irradiation

**
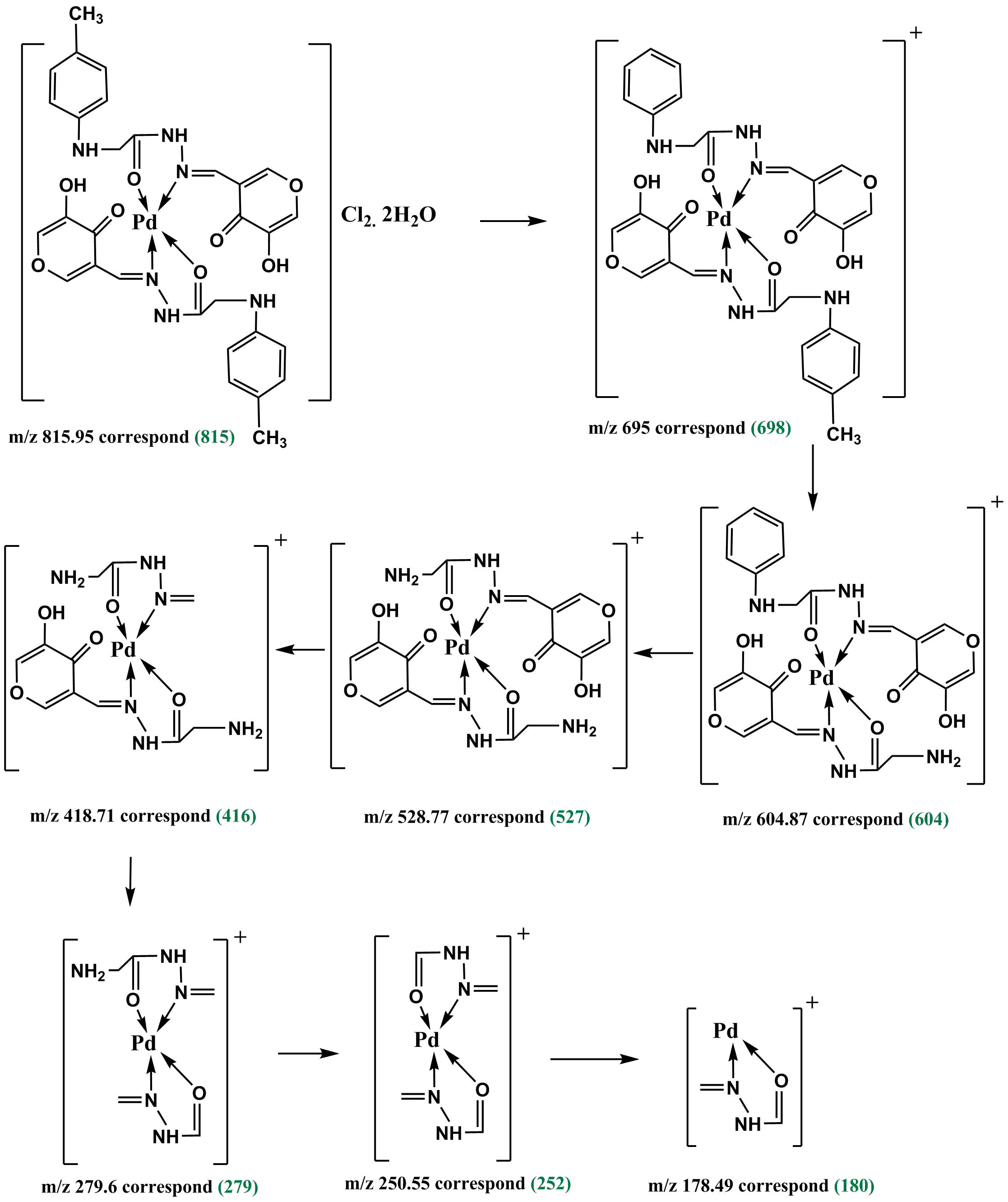
**

**Scheme S1:** Mass fragmentation of Pd(H_2_L)_2_]Cl_2_.2H_2_O complexes

**
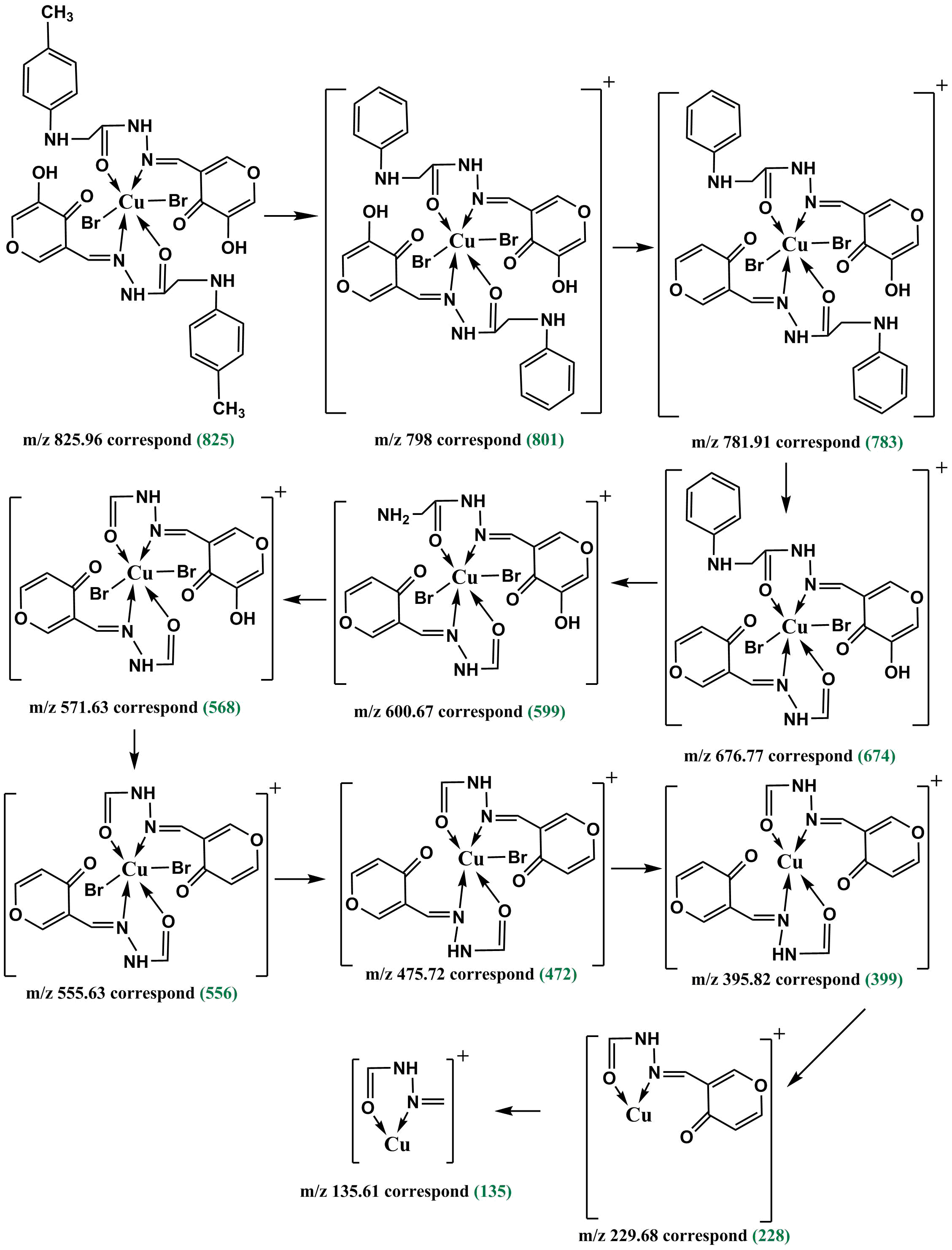
**

**Scheme S2:** Mass fragmentation of Cu(H_2_L)_2_Br_2_  complexes

**
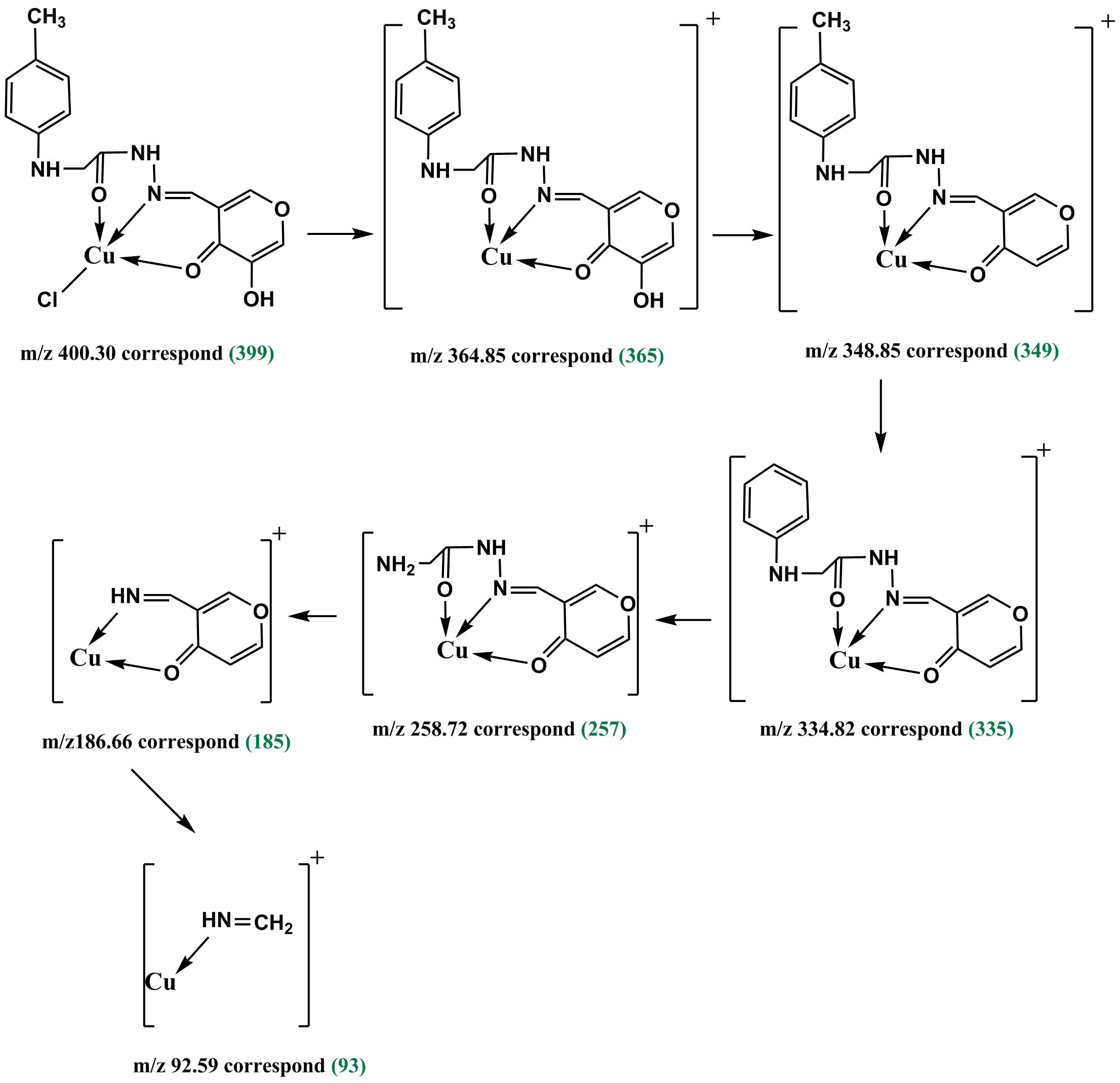
**

**Scheme S3:** Mass fragmentation of Cu(H_2_L)Cl complexes

Where:

Black color = the calculation value

Green color = the found value in the mass spectra figure
